# Dynamics of gut metabolome and microbiota maturation during early life

**DOI:** 10.1016/j.isci.2025.113596

**Published:** 2025-09-25

**Authors:** Anna-Katariina Aatsinki, Santosh Lamichhane, Heidi Isokääntä, Partho Sen, Matilda Kråkström, Marina Amaral Alves, Anniina Keskitalo, Eveliina Munukka, Hasse Karlsson, Laura Perasto, Minna Lukkarinen, Matej Oresic, Henna-Maria Kailanto, Linnea Karlsson, Leo Lahti, Alex M. Dickens

**Affiliations:** 1 Centre for Population Health Research, University of Turku and Turku University Hospital, Turku, Finland; 2 FinnBrain Birth Cohort Study, Turku Brain and Mind Center, Department of Clinical Medicine, University of Turku, Turku, Finland; 3 Turku Bioscience Centre, University of Turku and Åbo Akademi University, 20520 Turku, Finland; 4 Research Center for Infections and Immunity, Institute of Biomedicine, University of Turku, Turku, Finland; 5 Walter Mors Institute of Research on Natural Products, Federal University of Rio de Janeiro, Rio de Janeiro, Rio de Janeiro 21941-902, Brazil; 6 Department of Clinical Microbiology, Turku University Hospital, 20520 Turku, Finland; 7 Faculty of Medicine, Microbiome Biobank, University of Turku and Turku University Hospital, Turku, Finland; 8 Department of Psychiatry, University of Turku and Turku University Hospital, Turku, Finland; 9 Department of Pediatrics and Adolescent Medicine, Turku University Hospital and University of Turku, Turku, Finland; 10 School of Medical Sciences, Örebro University, 702 81 Örebro, Sweden; 11 Department of Public Health, University of Turku and Turku University Hospital, Turku, Finland; 12 Department of Child Psychiatry, Turku University Hospital and University of Turku, Turku, Finland; 13 Department of Life Technologies, University of Turku, 20014 Turku, Finland; 14 Department of Computing, University of Turku, 20014 Turku, Finland; 15 Department of Chemistry, University of Turku, 20520 Turku, Finland

**Keywords:** Gastroenterology, Pediatrics, Microbiology, Microbiome, Omics, Metabolomics

## Abstract

Early-life gut microbiome-metabolome crosstalk plays a crucial role in maintaining host physiology. The microbially produced metabolites often convey effects on host health and physiology. This study investigates the gut metabolites, including short-chain fatty acids (SCFAs), bile acids (BAs), and polar metabolites, and their relationship to gut microbiota composition in a birth cohort of 670 children. Samples were collected at 2.5 (*n* = 272), 6 (*n* = 232), 14 (*n* = 289), and 30 months (*n* = 157) of age.

We identified the trajectories of the fecal metabolome that relate to the maturation of the early-life gut microbiota. We found that prevalent gut microbial abundances were associated with microbial metabolite levels, particularly in 2.5-month-old infants. Here, the abundances of early colonizers, e.g., *Bacteroides, Escherichia*, and *Bifidobacterium*, were associated with microbial metabolites, especially secondary BAs, particularly in breastfed infants.

Our results suggest that early-life gut microbiota associates with changes in metabolome composition, particularly BAs, which may have physiological implications.

## Highlights

• Breastfeeding is the most dominant factor associated with metabolite levels
• SCFA concentrations, except acetic acid, increase during the first 30 months
• Generally, secondary BA increases, while taurine conjugated BA decreases
• Microbiota dominated by Bifidobacterium and Bacteroides had less conjugated BA

## Graphical abstract

**Figure:**
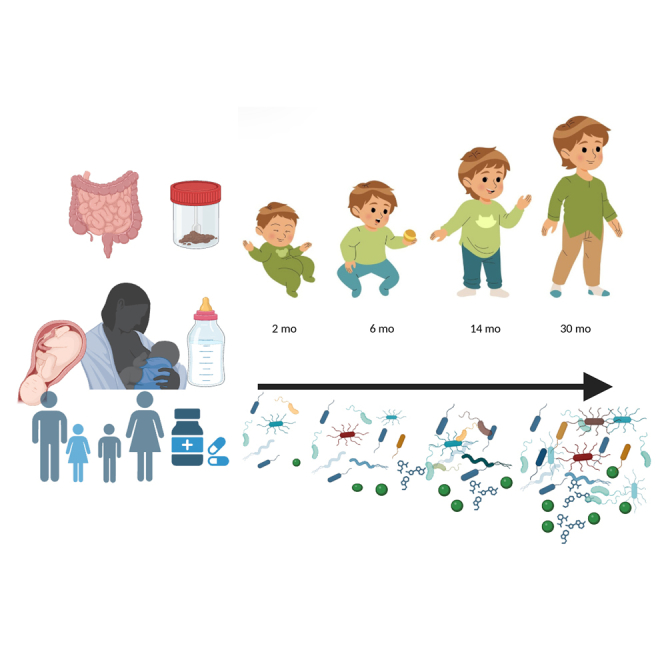

## Introduction

Human adult gut harbors an estimated average of 500–1000 species of microbes. The gut microbiome, which includes the by-products of microbes, as amino acids, vitamins, and organic acids, and the host interaction, is considered to be an “essential organ” within human beings.^1^^,^^2^^,^^3^ The process of gut microbiome colonization after birth has been intensively studied during the last decade.^4^^,^^5^^,^^6^ It has been established that members of *Bifidobacterium* and *Enterobacteriaceae* are typical in early infancy, whereas *Bacteroides* and *Ruminococcus* increase in abundance during later development, particularly when the diet diversifies^5^^,^^7^^,^^8^^,^^9^^,^^10^ Recent studies have shown that gut microbiome plays a crucial role in human health and disease, and its disturbances associate with many common diseases including inflammatory bowel disease,^11^ obesity,^12^ and various neurological and psychiatric disorders.^13^^,^^14^

Crosstalk between the gut microbiome and host metabolism is vital for maintaining human metabolic capacity.^15^ Many complex interactions between the gut microbiome and the host occur via enterohepatic circulation between the liver and the intestine, and the capacity for metabolite production begins already prenatally.^16^^,^^17^ As such, profiling fecal metabolites can provide an indirect functional readout of the gut microbiome composition. The metabolites can act as an intermediate phenotype mediating host-microbiome interactions.^18^ In fact, bidirectional interactions exist between the gut microbiome and metabolome.^19^ For example, the microbial biotransformation of bile acids (BAs) can regulate human physiology, and in turn, the overall host BA pool can control the microbial diversity.^20^ Intriguingly, a recent rodent study suggests that gut metabolome drives gut microbiota development and maturation.^21^ However, our understanding of early-life gut microbiota-metabolome maturation trajectories in humans is limited.^5^^,^^22^^,^^23^^,^^24^ Previous studies have shown short-chain fatty acid (SCFA) concentration increases by age, of which acetate typically plateaus first and remains relatively stable.^25^^,^^26^^,^^27^^,^^28^ Additionally, there are individual studies highlighting the developmental patterns in bile acids, untargeted metabolites, and aromatic amino acids,^27^^,^^29^ or study infant or child gut metabolites cross-sectionally.^30^ The existing literature also highlights that preterm birth associated with gut metabolite levels,^31^ there are age-related changes in the metabolite levels,^29^ and that early feeding causes variation in the gut metabolome composition.^32^^,^^33^^,^^34^^,^^35^ However, few studies have integrated different metabolomic assays and gut microbiota data in a longitudinal fashion in a general population-based birth cohort study. Although early feeding has been identified as an important factor causing variation in the gut metabolome in several studies,^33^^,^^34^^,^^35^ a comprehensive view on how and which early life factors associate with gut metabolome development is missing.

Here, we study how the early-life gut metabolome is associated with the maturation of gut microbiota. More specifically, we aimed to identify trajectories of fecal metabolome that may drive the maturation of early gut microbiota. We also explore how different early life factors, such as breastfeeding, are associated with gut metabolome and microbiota.

## Results

The study subjects are described in Table S1. We analyzed longitudinal metabolome, using both mass spectrometry based targeted and untargeted techniques, and microbiota in stool samples collected at 2.5 (*n* = 444 for microbiota, *n* = 272 for metabolome), 6 (*n* = 256 for microbiota, *n* = 232 for metabolome), 14 (*n* = 302 for microbiota and *n* = 289 metabolome), and 30 (*n* = 207 for microbiota and *n* = 157 for metabolome) months (mo) of age. The metabolomics dataset used for the analysis included identified metabolites from the following classes: short chain fatty acids (SCFA), bile acids (including taurine (tauro) and glycine (glyco) conjugated BAs), amino acids, carboxylic acids (mainly free fatty acids and other organic acids), hydroxy acids, phenolic compounds, alcohols, and sugar derivatives. There was no complete overlap between the timepoints: 37 children had microbiota data from all the timepoints, whereas two or three samples were available from 208 or 110 children, respectively.

### Fecal metabolites change during early development

**Figure 1:**
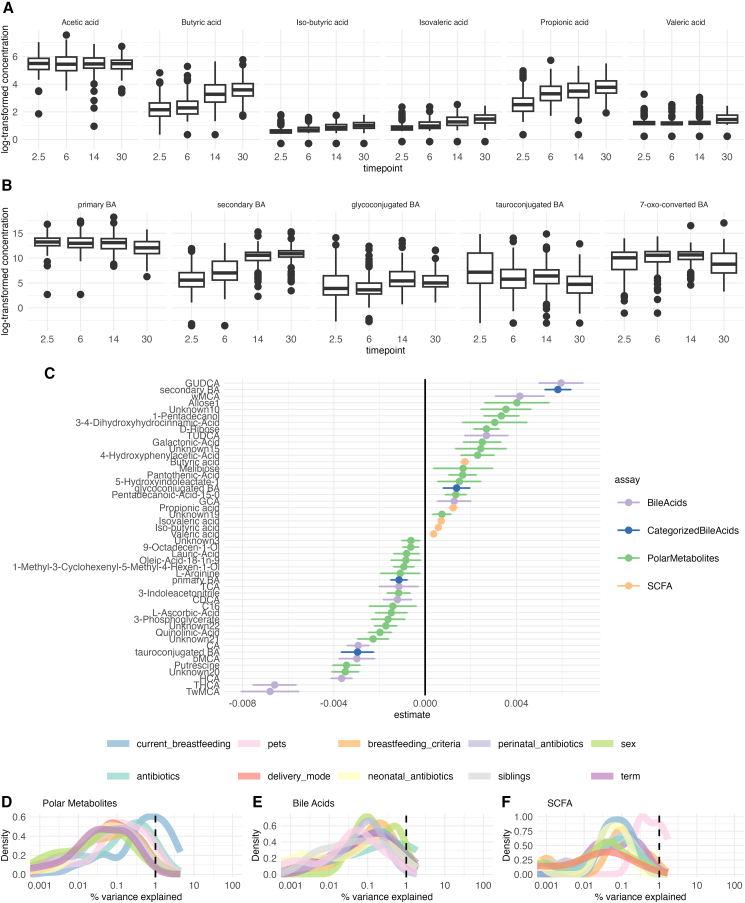
Metabolites varied in their age trends. SCFA tended to increase, while conjugated BA tended to decrease (A and B) The average changes in SCFAs and BAs concentrations were observed across different age groups. Each box in the plot shows the median (horizontal line), the interquartile range (box spanning the 25th to 75th percentiles), and whiskers extending to data points within 1.5 times the interquartile range from the quartiles. (C) Fixed effect-size (age coefficients) for individual metabolites as estimated from the linear mixed models, with metabolite concentration as the response variable, age as the fixed effect, and child as the random effect. Error bars represent 95% confidence interval- Lighter colors indicate lower concentration. Density plots showing explained variances (%) by total (D). polar metabolites, (E) Bas, and (F) SCFAs associated with the clinical and demographic factors.

First, we explored how the gut metabolome changes with age. As expected, age-related variation displayed the major effect on the gut metabolome. Most of the SCFAs, except for acetic acid, increased with age (Figure 1A). Individual BAs and polar metabolites showed no clear age-related patterns (Figure 1). Secondary BAs were positively, while, primary and tauroconjugated BAs remained negatively associated with age (Figures 1B and 1G) Glycoconjugated BAs were positively associated with age, however, this association attenuated when adjusting for breastfeeding (Figure S1). Some of the metabolites, including 5-hydroxyindoleacetate, 4-Hydroxyphenylacetic acid, and multiple unidentified polar metabolites that had a significant age trend, were attenuated when adjusting for breastfeeding associated with breastfeeding (Figure S1). Next, we also sought to explore the SCFA and BA trends in the subsample that had all the timepoints available (*n* = 37, Figure S2). We found that the trends were similar to those in the whole sample set.

### Breastfed children have lower concentrations of bile acids

In order to understand the overall contributions of various factors to gut metabolome, we performed variance analysis using variables previously shown to associate with gut microbiota maturation, i.e., breastfeeding, delivery mode, antibiotics intake, prenatal birth, biological sex assigned at birth, pet ownership, and having siblings. In general, demographic exposures explained on average < 1% of variance in polar metabolites, SCFAs, and BA concentrations (Figures 1D–1F).

**Figure 2:**
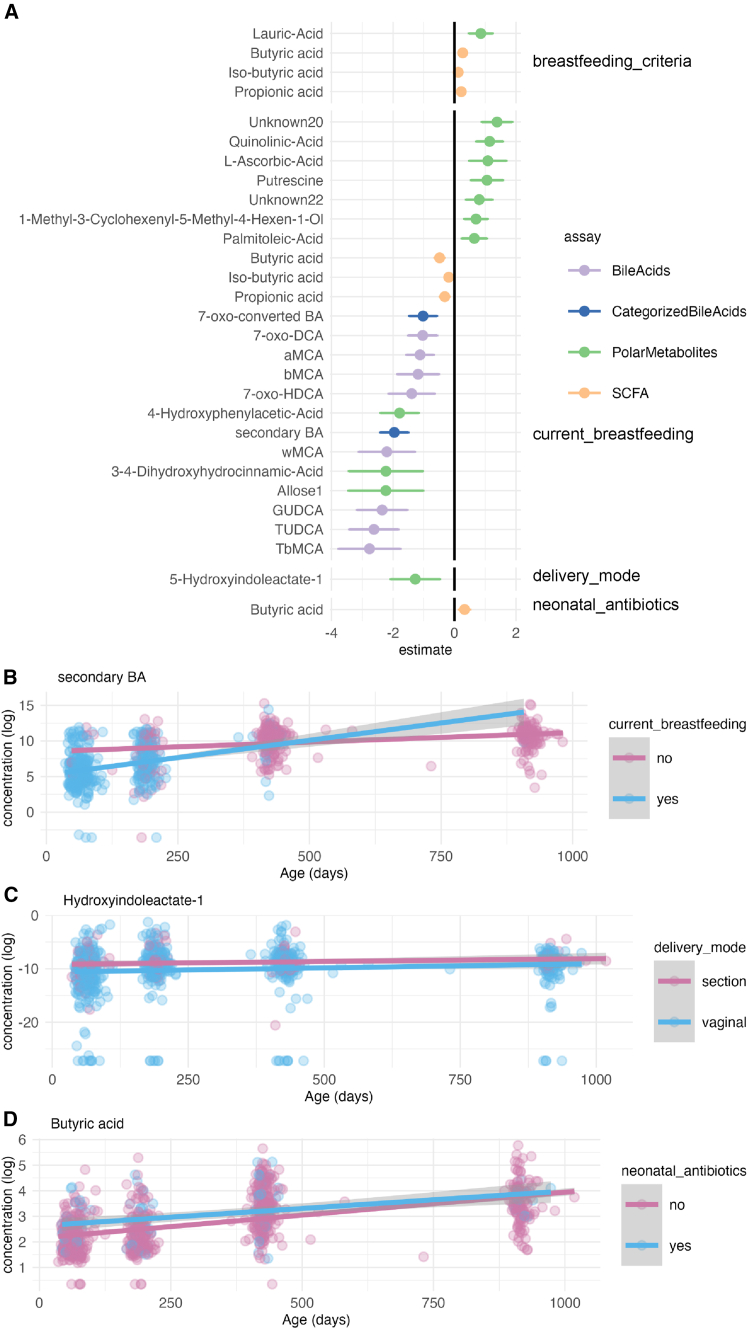
Out of a comprehensive list of background factors, breastfeeding associated with concentrations of multiple metabolites from different assays (A) Estimates for each demographic variable from a mixed model with the metabolite concentration as the dependent variable, demographic variable, and age as fixed effects, and child identity as a random effect. Error bars represent 95% confidence intervals. (B) Secondary BA concentrations were lower among breastfed infants in the 2.5-6-month timepoints. (C) Vaginally born infants had consistently lower concentration of hydroxyindoleacetate-1 across all timepoints. (D) Concentration of butyric acid was higher in infants who received antibiotic treatment in the neonatal period. (B–D) Gray area depicts a 95% confidence interval.

Next, to study in more detail how gut metabolites relate to demographic exposures, we implemented a linear mixed-effect model with metabolite concentration as the response variable, age and demographic variables as fixed effects, and child as a random effect. We found that breastfed infants had lower concentration of secondary and individual tauro- and glycoconjugated BAs, especially at an early age (Figures 2A and 2B; Figures S3 and S4).

Further, we investigated if the duration of exclusive breastfeeding was associated with metabolite concentrations in the 6, 14, and 30-month-olds (median 4.5 months, mean = 3.96, SD 1.95). We modeled the metabolite concentration by the duration of exclusive breastfeeding, age, and any current breastfeeding (fixed effects), and child identity (random effect). Specifically, the duration of exclusive breastfeeding was negatively associated with pinitol, lauric acid, ribonic acid, 1,2,3,4,5,6-hexatrimethylsilylinositol, 7-oxo-HDCA, propionic acid, and iso-butyric acid, whereas succinic acid was positively associated (q < 0.05).

Vaginal delivery was related to a lower concentration of hydroxyindoleacetate (Figure 1C), and exposure to intravenous antibiotics in the neonatal period was associated with a higher butyric acid concentration (Figure 1D). In the cross-sectional group comparison, vaginally born infants had a lower concentration of 7-oxo-converted BA at 14 months. The primary BAs at 14 months, and tauroconjugated BAs at 2.5 months were also lower. Likewise, breastfed infants had lower concentrations of secondary and primary BAs at 2.5 months. Having pets was positively associated with tauroconjugated BAs concentration at 14 months, whereas having siblings was positively associated with secondary BAs concentration at 6 and 14 months. It seems that factors related to optimal microbiota development, especially breastfeeding, associated with fecal metabolite concentrations.

### Infant microbiota shows a more diverse microbiota community type compared with toddlers

**Figure 3:**
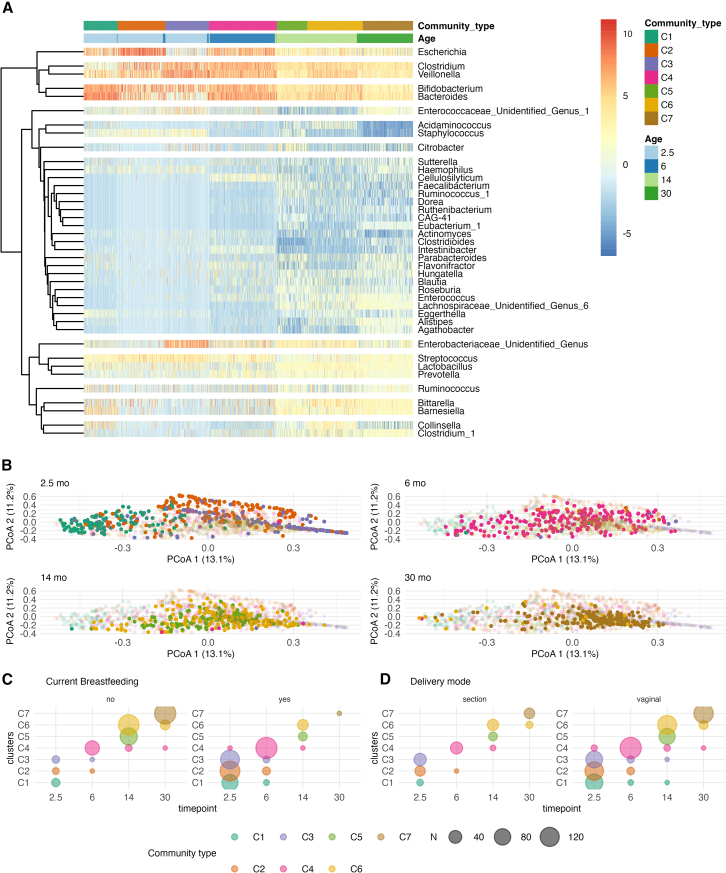
We stratified gut microbiota community composition into distinct community types with the Dirichlet Multinomial Mixture model, and linked the community types with breastfeeding and delivery mode (A) We identified seven community types. The breaks between rows are derived from the hierarchical clustering of clr-transformed abundances of genera with prevalence over 10% and abundance over 0.1%. Here, 50% height of the maximum dendrogram branch height is used to visualize clusters of taxa in the heatmap. (B) The community membership is indicated on the PCoA ordination. It seems that C7 was the most homogenous, as indicated by DMM theta (Table S4). The data points with a non-transparent color belong to the timepoint indicated above the figures, and the partially transparent points belong to other time points. The color represents the community type. (C and D) In a mixed model with the community type membership as the dependent variable, demographic variable, and age as fixed effects, and child identity as a random effect, (C) current breastfeeding and (D) delivery mode explained transition between community types.

Previous literature has suggested a successional development of infant gut microbiota taxonomic composition, and we wanted to confirm these patterns in our data.^5^^,^^36^ To examine the patterns of gut microbiota succession during early-life we performed Dirichlet Multinomial Mixture (DMM) model to identify gut microbiota community types and stratify the individuals accordingly. We identified 7 community types according to Laplace criteria when jointly analyzing the samples from all time points. The first timepoint was dominated by three community types, which were driven by the abundances of *Bacteroides* and *Bifidobacterium* (C1), *Escherichia* (C2), *Veillonella,* and an unidentified genus in *Enterobacriaceae* (C3), (Figure 2; Figure S5). The majority of the later timepoints were dominated by a single community type that were driven by *Bacteroides*, *Clostridium,* or *Veillonella* with differing proportions (C4-7, Figure 3; Figure S5).

Consistent with previous reports, the gut microbe community differed according to the background factors, including delivery mode and breastfeeding (Figure 3). Some additional trends were consistent with earlier reports but did not reach statistical significance. On the other hand, infant sex, having pets, the overall duration of exclusive breastfeeding, and intravenous neonatal or recent antibiotic intake were not associated with gut microbe community membership. When stratified by timepoint, delivery mode at 2.5 months (C1 3.3%, C2 15.1%, C3 29.4% of C-section born infants, Χ^2^ q < 0.005, Figure S6) and preterm delivery at 6 months (C1 50%, C2 89%, C3 67%, C4 98%, Figure S6) were enriched in a specific community type (Tables S2 and S3), whereas perinatal (2.5 months, 30 months) and recent antibiotic treatments (6 months, 30 months), siblings (2.5 months, 14 months) were not significant. In a mixed model with the community type membership as the dependent variable, demographic variable, and age as fixed effects, and child identity as a random effect, the vaginal delivery and current breastfeeding were negatively related to community type progression (Figures 2C and 2D).

### Microbiota alpha diversity and genera abundances are associated with fecal metabolites

**Figure 4:**
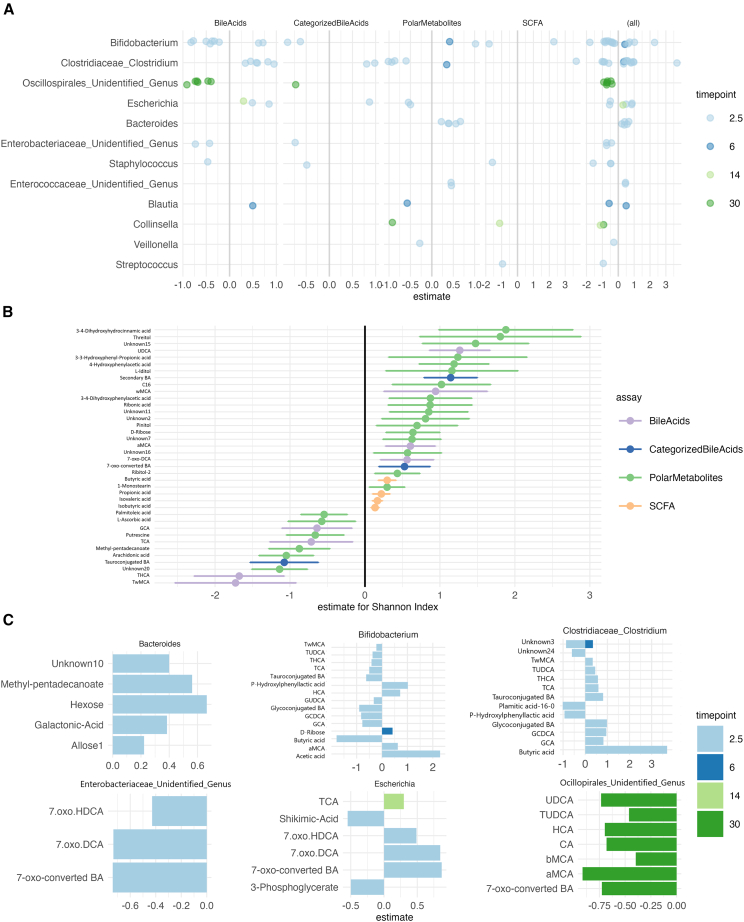
Gut microbiota composition associated with fecal metabolite concentrations, and the associations with genera differed based on age (A) Differential abundance analysis showed multiple associations between genus abundances and metabolites (ALDEx2). Only significant associations (q < 0.05) are visualized. *Bifidobacterium* (*n* = 22), *Clostridium* (*n* = 18), unidentified genus in Oscillaspirales (*n* = 11), *Bacteroides* (*n* = 9), *Escherichia* (*n* = 9) had most significant associations. (B) SCFA tended to positively correlate with alpha diversity, whereas individual polar metabolites and BAs correlated both negatively and positively. Error bars represent 95% confidence intervals. (C) Most significant associations between genera and metabolites were at 2.5 months time point. *Bifidobacterium* at 2.5 months associated negatively, and *Clostridium* at 2.5 months associated positively with conjugated BAs and butyric acid. *Streptococcus* was associated negatively with propionic acid. Unidentified genus in Oscillospiroles at 30 months associated negatively with multiple BAs, especially 7-oxo-converted and tauroconjugated BAs. Data is represented as the effect size derived from ALDEx2.

**Figure 5:**
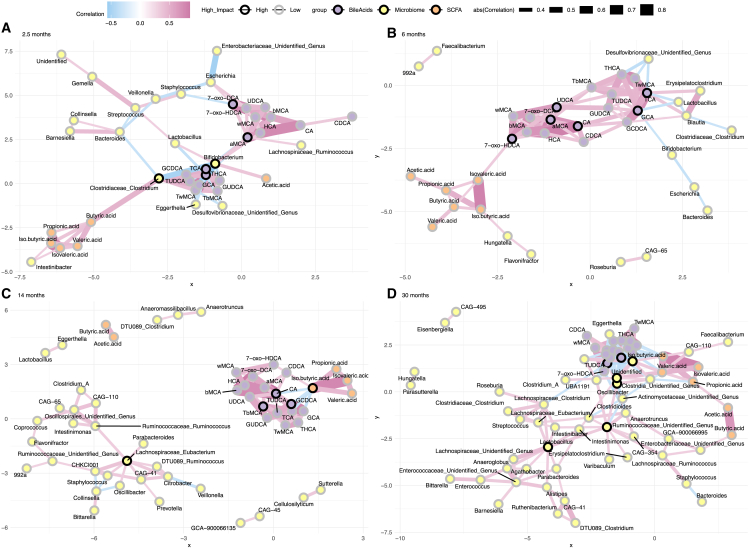
Networks of microbiome and metabolite intercorrelations are dependent on the age Spearman correlation was used here. Visually, the 30 months were dominated by microbial inter-correlations, whereas correlations with metabolites were limited. At 2.5 months, *Bifidobacterium* and Clostridium are both associated with bile acids and short-chain fatty acids. As expected, both SCFA and BA had strong correlations within the group. Black outer circle indicates “high impact,” i.e., nodes that have high degree and betweenness, and are in the top 25% in both. The color of the line indicates the Spearman correlation coefficient, and width of the line indicates the absolute strength of the correlation. (A) 2.5 months, (B) 6 months, (C) 14 months, (D) 30 months.

Next, we sought to determine whether the microbiota composition was associated with metabolome profiles. We found microbiota alpha diversity was correlated with multiple metabolite classes (Figure 5), in particular, SCFA concentration showed consistently positive associations with alpha diversity. The observed richness was also associated positively with SCFA concentration. Linear mixed model showed that THCA, TωMCA and several polar metabolites (Arachidonic acid, 2-Methylpentadecanoic acid, Putrescine) were negatively associated with Shannon Index, adjusted for age (fixed effect) and child identify (random effect) (q < 0.015, Figure 4), whereas butyric, propionic, isovaleric and iso-butyric acid, ωMCA, αMCA, UDCA, and other polar metabolite concentrations were positively associated with Shannon index when adjusted for age (*p* < 0.039, Figure 4). In addition, we found the Shannon index was positively associated with 7-oxo-converted BA concentrations (estimate = 0.3, 95%-CI 0.3–0.56, q = 0.047). In differential abundance testing, *Clostridium* and *Bifidobacterium* showed associations with butyric acid, P-hydrophenyllactic acid, and conjugated BAs in opposing directions (Figure S7). In 30-month-olds, unidentified genera in the Oscillospirales order associated negatively with BA, such as the 7-oxo-converted BA (Figure S8).

We performed a network analysis for each timepoint. There, the node and edge numbers were higher in the 30 months, and the density was highest in the first timepoint (Figure 5). Additionally, the degree distribution was more left-skewed in the 30 months compared with 6 and 14 months (Kolmogorov-Smirnov test, *p*-value 0.046 for 6 months, *p*-value 0.024 for 14 months). There were the most sub-communities in the 14 months, and it had the highest modularity score (Sub-communities 2.5 months *n* = 5, 6 months *n* = 7, 14 months *n* = 13, 30 months *n* = 10, 2.5 months modularity = 0.55, 6 months modularity = 0.53, 14 months modularity = 0.57, 30 months modularity = 0.37). Metabolites and genera with the highest degree and betweenness were different depending on the time point. For instance, *Bifidobacterium* and *Clostridium* had high degree and betweenness in the 2.5-month-olds, whereas Oscillaspirales and Ruminococcaceae had a high degree and betweenness in the 30-month-olds (Figure 5). Bile acids also had a high score, and CA was among the most connected metabolites in 6, 14, and 30 months (Figure 5).

### Microbiota community types associate with different levels of metabolites

Furthermore, we examined whether metabolite concentrations were different between community types. Community types showed different levels of fecal metabolites per timepoint, and the largest effect sizes were for TwMCA, TCA, THCA, GCA, as well as succinic acid and an unknown polar metabolite at 2.5 months. For all the above-mentioned BAs, C1 had a lower concentration compared with C2 and/or C3. Additionally, both glucoconjugated and tauroconjugated BA concentrations were lower in C1 at 2.5 months. At 14 months, butyric acid concentration was higher in C6 compared with C5. Additionally, at 30 months, C7 had higher concentrations of valeric acid, βMCA, succinic acid, and βMCA with a moderate effect size.

**Figure 6:**
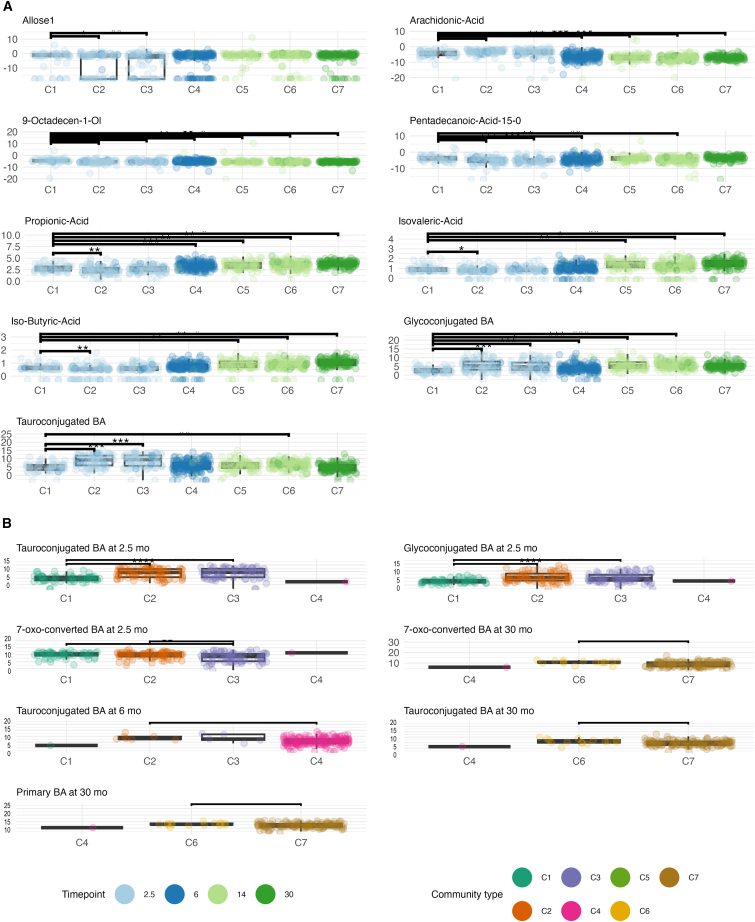
Community type showed different levels of metabolites (A) Several associations remained after adjusting for breastfeeding in the mixed model with the metabolite concentration as the dependent variable, community type, child age, and current breastfeeding as the fixed effects, and child identity as a random effect, color indicating the timepoint. (B) Community types at 2.5, 6, and 30 months had different levels of BA based on cross-sectional group comparison and post hoc testing. ∗q < 0.05 and q > 0.01, ∗∗q ≤ 0.01 and q > 0.001, ∗∗∗q ≤ 0.001. Each box in the plot shows the median (horizontal line), the interquartile range (box spanning the 25th to 75th percentiles), and whiskers extending to data points within 1.5 times the interquartile range from the quartiles.

The BAs TωMCA, THCA, TCA, GCA, and arachidonic acid showed a positive association with community type membership. Whereas multiple polar metabolites, UDCA, propionic acid, and branched SCFA showed negative associations with community type membership (Figure 6). Likewise, glycoconjugated and tauroconjugated were both positively associated with community types C2-C6 and C2, C3, and C6, respectively (FDR <0.05, C1 as reference, Figure 6). In addition, between-community type differences in SCFA and BA concentrations were similar in the subsample of subjects with the whole timeseries available (Figure S9).

### Interactions between breastfeeding, gut microbiota, and metabolites

Breastfeeding drives the microbiota maturation. We observed that breastfeeding showed the strongest associations with metabolite levels. Thus, we wanted to further explore the interactions between gut microbes’ abundances, metabolite levels, and breastfeeding. As the prevalent genera were driving the community types and they showed the most associations with metabolite levels, we studied how the interaction between prevalent genera and breastfeeding status associated with metabolite levels, with metabolite concentration as the dependent variable, age, and the interaction between any current breastfeeding and rclr-transformed abundance of genus as the fixed effects, and child identity as the random effect.

**Figure 7:**
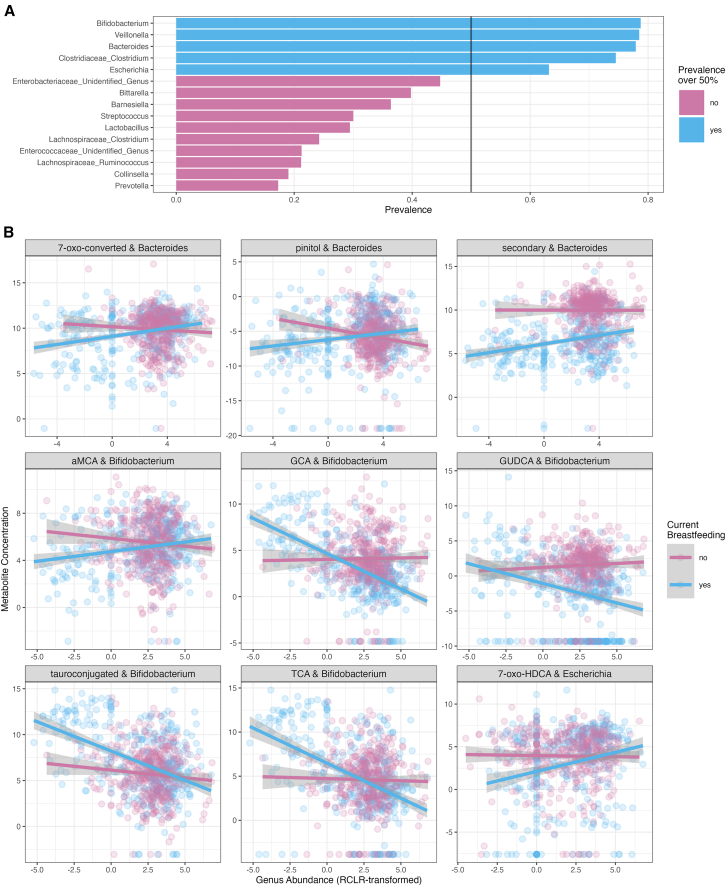
Prevalent genera abundances interaction with breastfeeding status associated with microbially metabolized metabolite concentrations (A) Only the five most prevalent taxa were observed in >50% of the study subjects, and those were selected for the interaction analyses. (B) *Escherichia, Bifidobacterium,* and *Bacteroides* showed significant interaction with breastfeeding. Scatterplots for significant interaction models. Gray areas depict 95% confidence intervals.

We observed that *Bifidobacterium* abundances were associated negatively with tauroconjugated BA concentration only in breastfed infants (Figure 7). On the other hand, *Bacteroides* was positively associated with secondary BA in the breastfed infants (Figure 7). Moreover, the less there is *Escherichia* in the gut microbiota of breastfed infants, the less there is 7-oxo-HDCA (Figure 7). Of the polar metabolites, *Bacteroides* abundances were positively associated with pinitol concentrations in the breastfed infants (Figure 7).

We further wanted to test if cumulative exclusive breastfeeding duration interacts similarly with prevalent genera abundances, with a model that had metabolite concentration as the dependent variable, age, any current breastfeeding, and the interaction between the duration of breastfeeding and rclr-transformed concentration of genus abundance as the fixed effects and child identity as the random effect. The interaction between exclusive breastfeeding duration and *Bacteroides* is associated with 7-oxo-converted BA and particularly 7-oxo-DCA (Figure S10). The interaction between exclusive breastfeeding duration and *Escherichia* abundances associated with 7-oxo-HDCA (Figure S10).

## Discussion

Gut microbiota undergoes successional development in early life,^36^ which is affected by factors such as breastfeeding and delivery mode.^5^ However, less is known about the development of fecal metabolites, which are important mediators of the physiological effects of the gut microbiota. Here, we showed in our population-based cohort that the fecal metabolome develops alongside the gut microbiota, and individual variation in microbiota is associated with the metabolome composition. Additionally, our observations suggest that breastfeeding, an important microbiota-modulating factor, is related to metabolite concentration depending on gut microbiota composition. This not only shows that the metabolome is related to microbiota development, but that common exposures may have individualized effects based on microbiota composition. We studied not only SCFA trends, but also BA and untargeted polar metabolites in our study in addition to showing links between gut metabolites and multiple early life exposures, thus extending the current understanding of the early life gut metabolome and associated factors.

SCFAs, except acetic acid, were systematically increased by age, and this might be explained by more complex microbiota and increased intake of indigestible fiber by age. This is in agreement with earlier studies, which suggest an increasing stool SCFA trend after birth^37^ with the exception of acetic acid. We observed no significant age-related increase for acetic acid, which might relate to the lack of very early sampling in our study. On the other hand, developmental patterns of BAs were more nuanced. Secondary BAs increased by age, whereas primary and tauroconjugated BAs decreased by age, which is partially in line with our previous findings.^8^ The decrease in BAs could be related to increased bile salt hydrolase (BSH) activity, potentially driven by increasing abundances of *Clostridium* and *Bacteroides.*^38^ Interestingly, glycoconjugated BAs were not increased by age when adjusting for breastfeeding. This could be explained by the observation that most of the infants in the first time point were breastfed, and thus harbored more *bifidobacteria*, which often have BSH enzymes with preference for glycine as a substrate over taurine.^39^ Thus, it may be that the *Bifidobacterium*-dominated microbiota is already capable of deconjugating glycine in earlier phases, which is further supported by our observation that *Bifidobacterium* was negatively associated with glycoconjugated BA concentration.

We observed that breastfeeding was related to lower abundances of butyric, iso-butyric, and propionic acid, which is in contrast to Brink et al. report.^39^ However, we noted a negative association between *Bifidobacterium* and butyric acid, which corroborates a finding by Nguyen et al. As noted by them, certain *Bifidobacterium* strains can compete for the same substrates as butyrate producers,^22^^,^^40^ and thus the strain-level variation between the studies may underline the discrepancies in the reports. Our data would suggest that if a breastfed infant has a lower *Bifidobacterium* or higher *Bacteroides* abundance, there is a concomitant higher concentration of microbially modified BAs. Both *Bacteroides* and *Bifidobacterium* are hallmark genera of the breastfed infants’ gut ecosystem, and those harbor differential capacity for BA metabolism. We acknowledge that strain level information is missing in our study. Notwithstanding that, we corroborate that secondary BA concentration was lower in breastfed infants,^41^ which might reflect slower acquisition of microbiota with BA metabolizing capacity.

In line with previous reports, we observed community typing in the gut microbiota that mostly aligned with age, reflecting typical colonization patterns in early life. Notable exception was the 2.5 months’ timepoint when most infants were breastfed, where three community types with either *Bifidobacterium* and *Bacteroides*, *Veillonella* and *Enterobacteriaceae* or *Escherichia* dominance were observed. *Bifidobacterium* and *Bacteroides* dominated community type was related to a lower rate of C-section, which aligns with the existing literature.^5^^,^^42^ Moreover, in addition to vaginal delivery, breastfeeding was related to a slower community type progression, which may indicate slower maturation of gut microbiota.^5^ Thus, our data would support the observation that the cessation of breastfeeding would result in the faster maturation of gut microbiota.

The differences in metabolite concentrations between community types in the 2.5-month-olds further elucidate the interaction between microbiota, factors affecting colonization, and metabolites. The *Bifidobacterium* and *Bacteroides* dominated community type, which had a higher proportion of vaginally born infants, was associated with a lower concentration of conjugated BAs than the two other major clusters at the first time point, most likely reflecting differences in BSH enzymatic activity. On the other hand, the *Bifidobacterium* and *Bacteroides* dominated community type had higher concentrations of propionic acid and branched SCFA iso-butyric and iso-valeric acids than the *Escherichia*-dominated community type, which may indicate the increased availability of protein for microbial fermentation. The difference may relate to variation in human milk composition,^43^ as no difference in breastfeeding was observed between community types dominating the first time point.

It is evident that breastfeeding is an essential factor in determining the microbiota. However, there is variation in the individual colonization patterns also in the breastfed infants, and we wanted to explore how the interaction between breastfeeding and prevalent taxa is associated with metabolite concentrations. Not surprisingly, conjugated BA concentrations were lower in the breastfed infants, the more they had *Bifidobacteria*. On the other hand, breastfed infants with high *Bacteroides* abundances had higher concentrations of secondary BAs. This indicates a complex interaction between early nutrition, early life microbiota, and microbially metabolized products. Thus, future focus on human milk components that potentially relate to the colonization patterns in microbiota when serving as substrates for microbial fermentation is warranted.

BAs participate in the regulation of inflammatory and metabolic processes via farnesoid X receptor and other bile acid-responsive receptors. For instance, secondary BAs, more abundant in breastfed infants with high *Bacteroides levels*, may inhibit pro-inflammatory processes in microglia,^44^ and they are also required to activate the vitamin D receptor to support optimal growth and development of adaptive immunity.^45^^,^^46^ Early-life microbiota-bile acid crosstalk may then participate in the programming of growth and later brain health. However, it is uncertain how exactly the complex feedback systems affect the physiological outcomes, since gut metabolites shape the postnatal gut microbiota composition,^21^ and for instance, tauroconjugated BAs metabolized by gut bacteria may, in feedback, inhibit BA synthesis via FXR antagonism.^47^

### Conclusions

First, we showed that SCFA concentrations, except acetic acid, and secondary BA increase, whereas tauroconjugated BA decrease within the first 30 months. Second, breastfeeding, among the background factors known to influence gut microbiota maturation, is associated with multiple metabolites. Interestingly, the secondary BA concentrations were lower in the breastfed infants. Third, we corroborated that gut microbiota shows successional maturation during the first 30 months of life. Fourth, we showed that prevalent gut microbe abundances are associated with metabolite levels, especially in the 2.5-month-olds. Finally, we demonstrate that the prevalent early colonizers *Bacteroides*, *Escherichia,* and *Bifidobacterium* abundances associate with the microbial metabolized Bas, especially in the breastfed infants. Alterations in early-life bile acid-microbiota crosstalk may, in future studies, prove an important mechanism in developmental programming of health. Breastfeeding and human milk composition are likely to be important moderators in the process.

### Limitations of the study

The main limitation of the study is the lack of longitudinal samples from all the participants, and we have partially distinct participants in the different time points. Although we can study group-level differences between developmental stages, the small sample size of participants with the full time series prevents us from detecting nuanced intra-individual dynamics of microbiota and metabolome. Moreover, although our study benefits from a large sample of children and a representative variation in breastfeeding and delivery mode, our sample collection time points do not extend to the neonatal time, nor was the sampling dense. This may have limited us to detect more nuanced patterns in the colonization and metabolome development. Additionally, our sample consisted mostly of infants and children who received some breastmilk, and we do not have an adequate sample size of exclusively formula-fed infants. The utilized 16S rRNA sequencing data provided important information on the overall microbiota profiles, but the results call for future studies focusing on gene-level differences in gut microbiota. Leveraging metagenomic sequencing in future studies will help to disentangle the role of BA metabolizing capacity in the developing gut microbiome. Moreover, more detailed data on early diet, such as analysis of human milk composition, may also help to describe the differences in microbiota composition and the functional output, especially in breastfed infants. Future integration of the reported exploratory findings to mechanistic models will help to elucidate the clinical potential related to inflammation^45^^,^^48^ and metabolic programming^38^^,^^45^^,^^48^ and metabolic programming.^45^^,^^48^ and metabolic programming.^45^^,^^48^ and metabolic programming.^45^^,^^48^ and metabolic programming.^45^^,^^48^ and metabolic programming.^45^^,^^48^ and metabolic programming.^45^^,^^48^ and metabolic programming.

## Resource availability

Due to national legislation on personal data protection and the rights of the study participants, the individual-level data cannot be made available online. The study subjects have given their consent after being informed that research data may be shared with research partners, that these partners are bound by confidentiality obligations, and that the participants will be informed of these partners on the research project website.

### Lead contact

Further information and requests for resources should be directed to and will be fulfilled by the Lead Contact, Linnea Karlsson (linnea.karlsson@utu.fi).

### Materials availability

Data can be shared with Research Agreement as part of research collaboration. Requests for collaboration can be sent to the Board of the FinnBrain Birth Cohort Study; please contact the lead contact mentioned above.

### Data and code availability

• The individual-level data cannot be shared openly due to national legislation and the rights of the study participants. This limits the generalizability of the findings.
• The R scripts for data analyses can be found in Zenodo (https://doi.org/10.5281/zenodo.14967319).
• The key resources table (supplement) presents the reagents and other items used in the study. However, this study did not generate new unique reagents.

## Acknowledgments

We want to thank all the participating families and the FinnBrain staff and assisting personnel. Turku Metabolomics Center and Biocenter Finland is acknowledged for the collaboration regarding fecal sample metabolomics. This work was supported by the “Inflammation in human early life: targeting impacts on life-course health” (INITIALISE) consortium funded by the Horizon Europe Program of the European Union under Grant Agreement 101094099.

Finnbrain Birth cohort Study (H.K.) has been funded by 10.13039/501100002341the Research Council of Finland (grant numbers 253270, 134950), 10.13039/501100004012Jane and Aatos Erkko Foundation, as well as 10.13039/501100004325Signe and Ane Gyllenberg Foundation. L.K. was funded by the 10.13039/501100002341Research Council of Finland (grant numbers 308176 and 325292), 10.13039/100010114Yrjö Jahnsson Foundation (6847, 6976), 10.13039/501100004325Signe and Ane Gyllenberg Foundation, Finnish State Grants for Clinical Research (P3654), 10.13039/100010125Jalmari and Rauha Ahokas Foundation, and 10.13039/100012107Waterloo Foundation (2110-3601). A.K.A. was supported by 10.13039/100010114Yrjö Jahnsson Foundation, Psychiatry Research Foundation, 10.13039/501100004756Emil Aaltonen Foundation, 10.13039/501100000942Brain Foundation, 10.13039/501100008413Instrumentarium Science Foundation, 10.13039/501100004325Signe and Ane Gyllenberg Foundation, Duodecim Finnish Medical Society, 10.13039/501100004037Juho Vainio Foundation, and 10.13039/501100002341Research Council of Finland (grant number 347640). H.I. had a grant from 10.13039/501100003125Finnish Cultural Foundation [no 00230482]. L.L. was supported by 10.13039/501100002341the Research Council of Finland (grant number 330887). E.M. was supported by the government research grant awarded to 10.13039/501100011797Turku University Hospital. A.D. has been funded by the 10.13039/100012107Waterloo Foundation and 10.13039/501100002341the Research Council of Finland (347924). “Inflammation in human early life: targeting impacts on life-course health” (INITIALISE) consortium funded by the Horizon Europe Program of the European Union under Grant Agreement 101094099 (to M.O., H.K., A.D.).

## Author contributions

Conception or design of the work: A.K.A., S.L., A.D., and L.L. Acquisition, analysis, or interpretation of data: L.K., H.K., E.M., H.M.K., H.I., A.K., L.P., M.L., M.O., A.D., A.K.A., S.L., L.L., and M.A.A. Drafting or substantial revision of the work: A.K.A., S.L., H.I., A.D., and L.L. All authors have approved the submitted revision.

## Declaration of interests

HMK is an employee of International Food and Fragnancies. EM was an employee of the Biocodex Finland. Other authors report no conflicts of interest.

## STAR★Methods

### Key resources table

**Table:** 

REAGENT or RESOURCE	SOURCE	IDENTIFIER
**Biological samples**		

Fecal	This paper	N/A

**Chemicals, peptides, and recombinant proteins**		

Propionic acid-d6	Toronto research chemicals	DRE-C16493010
Hexanoic acid-d3	Sigma Aldrich	489727-100 MG
All bile acids information can be found in our previous paper	N/A	Lamichhane et al.^8^
4,4-Dibromooctafluorobiphenyl	Sigma Aldrich	101990-1G
Acetic acid	Sigma Aldrich	84874.18
Propionic acid	Sigma Aldrich	8.00605.0100
Butyric acid	Sigma Aldrich	B103500-100 ML
Valeric acid	Sigma Aldrich	240370-5 ML
Hexanoic acid	Sigma Aldrich	21529-5 ML
Isobutyric acid	Sigma Aldrich	I1754-100 ML
Iso-valeric acid	Sigma Aldrich	129542-100 ML
N-Methyl-N-(trimethylsilyl) trifluoroacetamide	Sigma Aldrich	69478
Methoxyamine hydrochloride	Sigma Aldrich	226904
Alkane mix	Sigma Aldrich	04070-5 ML
Heptadecanoic acid	Sigma Aldrich	H3500-5G
valine-d8	Sigma Aldrich	486027
Glutamic acid-d5	Sigma Aldrich	616281
Hexane	Sigma Aldrich	34484-2.5L
Oleic acid	Sigma Aldrich	O1008-1G
Linoleic acid	Sigma Aldrich	L1376-1G
Decanoic acid	Sigma Aldrich	210409-5G
3-Hydroxybenzoic acid	Sigma Aldrich	H20008-5G
Octanoic acid	Sigma Aldrich	W279900
Lactic Acid	Sigma Aldrich	L6402-1g
Succinic acid	Sigma Aldrich	PHR1418-1G
2-Hydroxybutyric acid	Sigma Aldrich	220116-5G
Glycerol-3-phosphate	Sigma Aldrich	P8877
D-Fructose-6-phosphate	Sigma Aldrich	T37985-100 MG
Glyceraldehyde-3-phosphate	Sigma Aldrich	39705-1 ML
Fructose	Sigma Aldrich	F0127-500g
Ribose-5-phosphate	Sigma Aldrich	R7750
Glucose-6-phosphate	Sigma Aldrich	G7879-500 MG
L-Lysine	Sigma Aldrich	L5501-5G
L-Serine	Sigma Aldrich	S4500-1G
L-Threonine	Sigma Aldrich	T8625-1G
L-Ascorbic Acid	Sigma Aldrich	95209-50G
L-Phenylalanine	Sigma Aldrich	78019-25G
L-Proline	Sigma Aldrich	81709-10G
L-Valine	Sigma Aldrich	V0500-25G
L-glutamic acid	Sigma Aldrich	95436-100 MG
L-Arginine	Sigma Aldrich	11009-25G-F
L-Glutamine	Sigma Aldrich	G3126-100G
Glycine	Sigma Aldrich	500-46-50G
L-alanine	Sigma Aldrich	05129-25G
L-Asparagine	Sigma Aldrich	A0884-25G
L-Methionine	Sigma Aldrich	M9625-5G
L-(−)-Malic Acid	Sigma Aldrich	240176-50G
Fumaric acid	Sigma Aldrich	47910
5-Hydroxyindole-3-acetic acid	Sigma Aldrich	H8876
Indole-3-lactic acid	Sigma Aldrich	I5508
Indole-3-propionic acid	Sigma Aldrich	57400-5G-F
Phosphoenolpyruvic acid	Sigma Aldrich	P7127
L-Tryptophan	Sigma Aldrich	T0254-1G
L-aspartic acid	Sigma Aldrich	A9256-100G
Citric Acid monohydrate	Sigma Aldrich	71498-250G
1H-Indole-3-acetic acid (3-Indoleacetic acid)	Sigma Aldrich	45533
Ornithine	Sigma Aldrich	57197-100 MG
DL-Glyceraldehyde	Sigma Aldrich	G5001-500 MG
Arachidonic acid	Sigma Aldrich	A3611-100 MG
Cholesterol	Sigma Aldrich	C8667
3-Hydroxybutyric acid	Sigma Aldrich	166898-1G
Palmitic acid	Sigma Aldrich	P0500-10G
Stearic acid	Sigma Aldrich	S4751
L-Tyrosine	Sigma Aldrich	93829-25G
Cysteine	Sigma Aldrich	168149-2.5G
Taurine	Sigma Aldrich	T0625-10G
Oxalacetate	Sigma Aldrich	O4126
Homocysteine	Sigma Aldrich	44925
L-Leucine	Sigma Aldrich	L8912-25G
AMPure XP magnetic bead	Beckman Coulter	A63880
Midori green, advance DNA stain	Nippon genetics	MG04
Generuler 100 bp DNA ladder	Thermo Scientific	SM0243
DNA Gel Loading Dye (6X)	Thermo Scientific	R0611
V4 primers: forward, reverse, index and read primers	biomers.net	custom-made, Rintala et al.^49^

**Critical commercial assays**		

GXT stool extraction kit	HAIN life science	12.06.02
Qubit dsDNA High Sensitivity Assay kit	Thermo Fisher Scientific	Q32854
KAPA high fidelity PCR kit with dNTPs	Roche	KK2102
Illumina MiSeq Reagent Kit v3 (600-cycle)	Illumina	MS1023003
Illumina Nextera XT Index Kit	Illumina	FC-131-1002
PhiX Control kit V3	Illumina	FC-110-3001

**Deposited data**		

Raw and analyzed data	This paper	Data can be shared with Research Agreement as part of research collaboration. Requests for collaboration can be sent to the Board of the FinnBrain Birth Cohort Study

**Software and algorithms**		

All bile acids software, described in our previous paper Lamichhane et al.^8^	N/A	N/A
All SCFA were quantified using MassHunter Quantatitive MS analysis v B0.9.00	Agilent	N/A
All polar metabolites were identified and quantified using Leco Chromatof V4.3	Leco	N/A

**Other**		

Analytical R Scripts∗	This Paper	https://doi.org/10.5281/zenodo.14967319

∗Analytical R Scripts: https://zenodo.org/records/14967319.

### Experimental model and subject details

The study subjects are children from the FinnBrain Cohort Study^50^ that is a general population birth cohort study located in the southwestern Finland. The FinnBrain Birth Cohort Study recruited families with sufficient fluency in Finnish or Swedish, and normal 1st trimester ultrasound examination. A subset of the cohort participated in the study visits, and there were no exclusion criteria for the collection of fecal samples. The initial recruitment took place between December 2011 and April 2015, and fecal samples were collected from May 2013 to May 2018. The fecal samples were collected from the children by the parents according to written and oral instructions at 2.5, 6, 14 and 30 months postpartum. The samples were collected in plastic tubes, and parents were instructed to store the sample in a refrigerator, and bring the sample to the laboratory within 24 h. The samples were processed in the Medical Microbiology laboratory of the Research Center for Infections and Immunity, University of Turku. The sample collection time was reported.

Clinical data used in the study were collected with parental reports during and after pregnancy at 14, 24, 34 gestational weeks, 3, 6, 12, and 24 months postpartum and during study visits (2.5, 6, 14, and 30 months). Likewise, the data on maternal pre-pregnancy body mass index (BMI; kg/m2), duration of gestation as well as mode of delivery (caesarian section vs. vaginal) were collected from National Birth Registry provided by the National Institute for Health and Welfare of Finland (www.thl.fi). The information on maternal perinatal and infant neonatal intravenous antibiotic intake was collected from the hospital records. Breastfeeding was categorized in two ways: 1) any current breastfeeding (yes vs. no); 2) exclusive breastfeeding at least 4 months and partial breastfeeding for at least 6 months (breastfeeding_criteria, yes vs. no).

Ethical issues have been considered and there is a research permit for the project. FinnBrain has a permit from the Ethics Committee of the the wellbeing services county of Southwest Finland (ETMK: 57/180/2011), which has approved Cohort profile and research protocol (Karlsson et al. 2018). FinnBrain parents have signed a consent form about their children’s participation in research and given permission to use their samples for scientific purposes. Samples went through the laboratory process anonymously with research code to protect participants’ privacy. STORMS guideline was used for reporting the methods and materials (Table S5).

### Method details

#### Metabolome analysis

The BAs were measured in fecal samples as described previously.^19^ Only samples frozen within 24 h of sample collection were included in the metabolome analyses. The order of the samples was randomized before sample preparation. Two aliquots (50 mg) of each fecal sample were weighed. An aliquot was freeze-dried prior to extraction to determine the dry weight. The second aliquot was homogenized by adding homogenizer beads and 20 μL of water for each mg of dry weight in the fecal sample, followed by samples freezing to at least −70°C and homogenizing them for 5 min using a bead beater. The BAs analyzed were Litocholic acid (LCA), 12-oxo-litocholic acid (12-oxo-LCA), Chenodeoxycholic acid (CDCA), Deoxycholic acid (DCA), Hyodeoxycholic acid (HDCA), Ursodeoxycholic acid (UDCA), Dihydroxycholestanoic acid (DHCA), 7-oxo-deoxycholic acid (7-oxo-DCA), 7-oxo-hyocholic acid (7-oxo-HCA), Hyocholic acid (HCA), β-Muricholic acid (b-MCA), Cholic acid (CA), Ω/α-Muricholic acid (w/a-MCA), Glycolitocholic acid (GLCA), Glycochenodeoxycholic acid (GCDCA), Glycodeoxycholic acid (GDCA), Glycohyodeoxycholic acid (GHDCA), Glycoursodeoxycholic acid (GUDCA), Glycodehydrocholic acid (GDHCA), Glycocholic acid (GCA), Glycohyocholic acid (GHCA), Taurolitocholic acid (TLCA), Taurochenodeoxycholic acid (TCDCA), Taurodeoxycholic acid (TDCA), Taurohyodeoxycholic acid (THDCA), Tauroursodeoxycholic acid (TUDCA), Taurodehydrocholic acid (TDHCA), Tauro-α-muricholic acid (TaMCA), Tauro-β-muricholic acid (TbMCA), Taurocholic acid (TCA), Trihydroxycholestanoic acid (THCA) and Tauro-Ω-muricholic acid (TwMCA). BAs were extracted by adding 40 μL fecal homogenate to 400 μL crash solvent (methanol containing 62,5 ppb each of the internal standards LCA-d4, TCA-d4, GUDCA-d4, GCA-d4, CA-d4, UDCA-d4, GCDCA-d4, CDCA-d4, DCA-d4 and GLCA-d4) and filtering them using a Supelco protein precipitation filter plate. The samples were dried under a gentle flow of nitrogen and resuspended using 20 μL resuspenstion solution (Methanol:water (40:60) with 5 ppb Perfluoro-*n*-[13C9]nonanoic acid as in injection standard). Quality control (QC) samples were prepared by combining an aliquot of every sample into a tube, vortexing it and preparing QC samples in the same way as the other samples. Blank samples were prepared by pipetting 400 μL crash solvent into a 96-well plate, then drying and resuspending them the same way as the other samples. Calibration curves were prepared by pipetting 40 μL of standard dilution into vials, adding 400 μL crash solution and drying and resuspending them in the same way as the other samples. The concentrations of the standard dilutions were between 0.0025 and 600 ppb.

The LC separation was performed on a Sciex Exion AD 30 (AB Sciex Inc., Framingham, MA) LC system consisting of a binary pump, an autosampler set to 15°C and a column oven set to 35°C. A waters Aquity UPLC HSS T3 (1.8 μm, 2.1 × 100 mm) column with a precolumn with the same material was used. Eluent A was 0.1% formic acid in water and eluent B was 0.1% formic acid in methanol. The gradient started from 15% B and increased to 30% B over 1 min. The gradient further increased to 70% B over 15 min. The gradient was further increased to 100% over 2 min. The gradient was held at 100% B for 4 min then decreased to 15% B over 0.1 min and re-equilibrated for 7.5 min. The flow rate was 0.5 mL/min and the injection volume was 5 μL.

The mass spectrometer used for this method was a Sciex 5500 QTrap mass spectrometer operating in scheduled multiple reaction monitoring mode in negative mode. The ion source gas1 and 2 were both 40 psi. The curtain gas was 25 psi, the CAD gas was 12 and the temperature was 650°C. The spray voltage was 4500 V. Data processing was performed on Sciex MultiQuant.

##### Quantification of SCFA

We adapted and modified the targeted SCFA analysis from previous work.^51^ Fecal samples were homogenized by adding water (10 μL per mg of dry weight as determined for the BA analysis) to wet feces, the samples were homogenized using a bead beater. Analysis of SCFA was performed on fecal homogenate (50 μL) crashed with 500 μL methanol containing internal standard (propionic acid-d6 and hexanoic acid-d3 at 10 ppm). Samples were vortexed for 1 min, followed by filtration using 96-Well protein precipitation filter plate (Sigma-Aldrich, 55263-U). Retention index (RI, 8 ppm C10-C30 alkanes and 4 ppm 4,4-Dibromooctafluorobiphenyl in hexane) was added to the samples. Gas chromatography (GC) separation was performed on an agilent 5890B GC system equipped with a Phenomenex Zebron ZB-WAXplus (30 m × 250 μm × 0.25 μm) column a short blank pre-column (2 m) of the same dimensions was also added. A sample volume of 1 μL was injected into a split/splitless inlet at 285°C using split mode at 2:1 split ratio using a PAL LSI 85 sampler. Septum purge flow and split flow were set to 13 mL/min and 3.2 mL/min, respectively. Helium was used as carrier gas, at a constant flow rate of 1.6 mL/min. The GC oven program was as follows: initial temperature 50°C, equilibration time 1 min, heat up to 150°C at the rate of 10 °C/min, then heat at the rate of 40 °C/min until 230°C and hold for 2 min. Mass spectrometry was performed on an Agilent 5977A MSD. Mass spectra were recorded in Selected Ion Monitoring (SIM) mode. The detector was switched off during the 1 min of solvent delay time. The transfer line, ion source and quadrupole temperatures were set to 230, 230°C and 150°C, respectively. Dilution series of SCFA standards of acetic, propionic, butyric, valeric, hexanoic acid, isobutyric, and iso-valeric acid were prepared in concentrations of 0.1, 0.5, 1, 2, 5, 10, 20, 40, and 100 ppm for the construction of standard curves for quantification.

##### Analysis of polar metabolites

Polar metabolites were extracted in methanol. The method was adapted from the method used by Lamichhane et al.^8^ Fecal homogenates (60 μL) were diluted with 600 μL methanol crash solvent containing internal standards (heptadecanoic acid (5 ppm) valine-d8 (1 ppm) and glutamic acid-d5 (1 ppm)). After precipitation the samples were filtered using Supelco protein precipitation filter plates. One aliquot (50 μL) was transferred to a shallow 96-well plate to create a QC sample. The rest of the sample volume was dried under a gentle stream of nitrogen and stored in −80°C until analysis. After thawing the samples were again dried to remove any traces of water. Derivatization was carried out on a Gerstel MPS MultiPurpoe Sampler using the following protocol: 25 μL methoxamine (20 mg/mL) was added to the sample followed by incubation on a shaker heated to 45°C for 60 min. N-Methyl-N-(trimethylsilyl) trifluoroacetamide (25 μL) was added followed by incubation (60 min). After that, 25 μL retention index was added, the sample was allowed to mix for 1 min followed by injection. The automatic derivatization was carried out using the Gerstel maestro 1 software (version 1.4).

Gas chromatographic (GC) separation was carried out on an Agilent 7890B GC system equipped with an Agilent DB-5MS (20 m x 0.18 mm (0.18 μm)) column. A sample volume of 1 μL was injected into a split/splitless inlet at 250°C using splitless mode. The system was guarded by a retention gap column of deactivated silica (internal dimensions 1.7 m, 0.18 mm, PreColumn FS, Ultimate Plus Deact; Agilent Technologies, CA, USA). Helium was used as carrier gas at a flow rate of 1.2 mL/min for 16 min followed by 2 mL/min for 5.75 min. The temperature program started at 50°C (5 min), then a gradient of 20 °C/min up to 270°C was applied and then finally a gradient of 40°/min to 300°C, where it was held stable for 7 min. The mass spectrometry was carried out on a LECO Pegasus BT system (LECO). The acquisition delay was 420 s. The acquisition rate was 16 spectra/sec. The mass range was 50–500 m/z and the extraction frequency was 30 kHz. The ion source was held at 250°C and the transferline heater temperature was 230°C. ChromaTOF software (version 5.51) was used for data aquisition. The samples were run in 9 batches, each consisting of 100 samples and a calibration curve. In order to monitor the run a blank, a QC and a standard sample with a known concentration run between every 10 samples. Between every batch the septum and liner on the GC were replaced, the precolumn was cut if necessary and the instrument was tuned.

The retention index was determined with ChromaTOF using the reference method function. For every batch a reference file was created. The reference file contained the spectras and approximate retention times of the alkanes from C10 to C30 as determined manually). A reference method was implemented for every sample in order to determine the exact retention time of the alkanes. Text files with the names and retention times of the alkanes were then exported and converted to the correct format for MSDIAL using an in-house R script. The samples were exported from ChromaTOF using the netCDF format. After this they were converted to abf files using the abfConverter software (Reifycs). Untargeted data processing was carried out using MSDIAL (version 4.7). The minimum peak height was set to an amplitude of 1000, the sigma window value was 0.7 and the EI spectra cut off was 10. The identification was carried out using retention index with the help of the GCMS DB-Public-kovatsRI-VS3 library provided on the MSDIAL webpage. A separate RI file was used for each sample. The RI tolerance was 20 and the m/z tolerance was 0.5 Da the EI similarly cut off was 70%. The identification score cut off was 70% and retention information was used for scoring. Alignment was carried out using the RI with an RI tolerance of 10. The EI similarity tolerance was 60%. The RI factor was 0.7 and the EI similarity factor was 0.5. The results were exported as peak areas and further processed with excel. In excel the results were normalized using heptadecanoic acid as internal standard and the features with a coefficient of variance of less than 30% in QC samples were selected. Further filtering was carried out to remove alkanes and duplicate features. The IDs of the features which passed the CV check were further checked using the Golm Metabolome Database.

#### Microbiota analysis

##### DNA extraction and sample processing

The samples were divided into cryotubes and frozen in −80C within 2 days after arriving at the laboratory. Samples were kept at +4C before freezing. Only samples that were frozen within 48 h of sample collection were sequenced. Sample volume for DNA extraction was approximately 100 mg. Lysis buffer was added 1 mL, and the samples were homogenized with glass beads 1000 rpm/3 min. The samples were centrifuged at high speed (>13000 rpm) for 5 min. The lysate (800 μL) was then transferred to tubes and the extraction proceeded according to the manufacturer’s protocol. DNA was extracted using a semi-automatic extraction instrument Genoxtract with DNA stool kit (HAIN life science, Germany). DNA yields were measured with Qubit fluorometer using Qubit dsDNA High Sensitivity Assay kit (Thermo Fisher Scientific, USA). The DNA extraction and sequencing was performed at the University of Turku.

##### 16S ribosomal RNA (rRNA) amplicon sequencing

Bacterial community composition was determined by sequencing the V4 region of 16S rRNA gene using Illumina MiSeq platform (Illumina, USA). The sequence library was constructed with an in-house developed protocol where amplicon PCR and index PCR were combined.

The DNA samples were diluted in PCR grade water to 10 ng/μL concentration prior to library PCR. PCR was performed with KAPA HiFi High Fidelity PCR kit with dNTPs (Roche, USA). Reverse and forward primers included in-house modifications verified by Rintala et al.^49^ The forward and reverse primer sequences were 5′-AATGAT-ACGGCGACCACCGAGATCTACAC -i5- TATGGTAATT -GT-GTGCCAGCMGCCGCGGTAA-3′ and 5′-CAAGCAGAAGACGGCATACGAGAT -i7- AGTCAGTCAG-GC-GGACTACHVGGGTWTCTAAT-3′, respectively, where i5 and i7 indicate the sample specific indexes. After PCR, 5 μL of the product was analyzed with 1.5% TBE agarose gel (100 V, 1 h 15 min). PCR products were purified with AMPure XP magnetic beads (Becman Coulter, USA). The DNA concentrations of the purified samples were measured with Qubit fluorometer using Qubit dsDNA High Sensitivity Assay kit (Thermo Fisher Scientific, USA), after which the samples were mixed in equimolar concentration into a 4 nM library pool. The library pool was denatured, diluted to a concentration of 4 pM and a denaturized PhiX control (Illumina, USA) was added. The sequencing was performed with Illumina MiSeq Reagent kit v3 (600 cycles) on MiSeq system with 2x 250 base pair (bp) paired ends following the manufacturer’s instructions. Positive control (DNA 7-mock standard) and negative control (PCR grade water) were included in library preparation and sequencing runs (Figures S11–S14).

DADA2-pipeline (version 1.14) was used to preprocess the 16S rRNA gene sequencing data to infer exact amplicon sequence variants (ASVs).^52^ The reads were truncated to length 225 and reads with more than two expected errors were discarded (maxEE = 2). SILVA taxonomy database (version 138)^53^^,^^54^ and RDP Naive Bayesian Classifier algorithm^55^ were used for the taxonomic assignments of the ASVs. Library sizes for all timepoints are shown in the Figure S15.

### Quantification and statistical analysis

The data analyses were performed with R version 4.2.0 with packages including *phyloseq*, *mia*, *vegan, DirichletMultinomial* and *lme*. Heatmaps were created with the *pheatmap* R package. Shannon Index and observed richness were used as alpha diversity indices and those were calculated with *mia* package from the untransformed ASV-table, i.e., count assay. Metabolite concentrations were log-transformed with a pseudocount (minimum value/2). Dirichlet Multinomial Mixture Model (DMM) with the rarified (minimum read count 10000), genus-level count data were used to identify community types in the microbiota data.^56^ The optimal number of community types was determined by the Laplace criteria.

Factor analysis, the relative contribution of a clinical/demographic factor toward the total variance of the metabolite classes were estimated by fitting a linear regression model. The total metabolite concentrations of a particular class were regressed to a clinical/demographic factor of interest, and median marginal coefficient of determination (R2) and % of explained variance were estimated. Factor analysis was performed using the *scater* package in R.

Wilcoxon test was used to test metabolite concentration difference between groups (such as breastfed and non-breastfed). Chi-square test was used to test difference in community type proportions between timepoints and groups (such as breastfed and non-breastfed). Kruskal-Wallis test with Dunn’s posthoc test were used to test metabolite concentrations differences between timepoints. Linear mixed models with child ID as random effect and sampling age as fixed effect were used to study i. metabolite age-trends, ii. association between metabolite concentrations and demographic factors, iii., association between microbiota community type membership and demographic factors, iv. associations between metabolite concentrations and microbiota community type membership, and v. association between metabolite concentrations and the interaction with breastfeeding and rclr-transformed prevalent genus abundances as breastfeeding has been shown to drive the microbiota maturation.^5^ Genera observed in >50% of the study subjects were categorized as prevalent. Package lme4 was used to check for model singularity, and nlme was used for running the mixed model. The clr-module from the ALDEx2 was used for the differential abundance analysis.^57^ Variance explained in the metabolome assays by demographic factors was calculated with the package *scater*.^58^ *p*-values were adjusted for multiple testing with Benjamini-Hochberg procedure.

## Supplemental information

Document S1. Figures S1–S15 and Tables S2–S4

Table S1. Sample characteristics, related to Figure 1

Table S5. STORMS checklist
