# Supplementary material for: Dynamics of gut metabolome and microbiota maturation during early life
Source: iScience. 2025 Sep 25;28(11):113596. doi: 10.1016/j.isci.2025.113596 (PMC12636393; doi:10.1016/j.isci.2025.113596)
Supplement: Document S1. Figures S1–S15 and Tables S2–S4 [file mmc1.pdf]

iScience, Volume *28*

Supplemental information

Dynamics of gut metabolome and microbiota maturation during early life

Anna-Katariina Aatsinki, Santosh Lamichhane, Heidi Isokääntä, Partho Sen, Matilda Kråkström, Marina Amaral Alves, Anniina Keskitalo, Eveliina Munukka, Hasse Karlsson, Laura Perasto, Minna Lukkarinen, Matej Oresic, Henna-Maria Kailanto, Linnea Karlsson, Leo Lahti, and Alex M. Dickens

Dynamics of Gut Metabolome and Microbiota Maturation during Early Life

Anna-Katariina Aatsinki[1,2]#, Santosh Lamichhane[3,4]#, Heidi Isokääntä[1,2,4]#, Partho Sen[3], Matilda Kråkström[3], Marina Amaral Alves[3,5], Anniina Keskitalo[5], Eveliina Munukka[6], Hasse Karlsson[1,2,7], Laura Perasto[1,2], Minna Lukkarinen[1,2,9], Matej Oresic[3,10,11], Henna-Maria Kailanto[1,2], Linnea Karlsson[1,2,8], Leo Lahti[12], Alex M Dickens[3,13*]

Supplementary figures and pages.

FIGURE S 1ESTIMATES AND 95 % CONFIDENCE INTERVALS FOR METABOLITES ASSOCIATED WITH AGE WHEN ADJUSTING FOR CURRENT BREASTFEEDING, RELATED TO FIGURE 1. ERROR BARS REPRESENT 95% CONFIDENCE INTERVALS. 3

FIGURE S 2 METABOLITE AGE-TRENDS IN THE SUBSAMPLE OF CHILDREN WITH THE WHOLE TIMESERIES, RELATED TO FIGURE 1. A., B. EACH BOX IN THE PLOT SHOWS THE MEDIAN (HORIZONTAL LINE), AND THE INTERQUARTILE RANGE (BOX SPANNING THE 25TH TO 75TH PERCENTILES). THE UPPER WHISKER EXTENDS FROM THE HINGE TO THE HIGHEST VALUE THAT IS WITHIN 1.5 * IQR OF THE HINGE, WHERE IQR IS THE INTER-QUARTILE RANGE. THE LOWER WHISKER EXTENDS FROM THE HINGE TO THE LOWEST VALUE WITHIN 1.5 * IQR OF THE HINGE. C. ERROR BARS REPRESENT 95% CONFIDENCE INTERVALS. 4

FIGURE S 3 SECONDARY BILE ACID CONCENTRATIONS IN BREASTFED AND NON-BREASTFED CHILDREN PER TIMEPOINT, RELATED TO FIGURE 2. EACH BOX IN THE PLOT SHOWS THE MEDIAN (HORIZONTAL LINE), AND THE INTERQUARTILE RANGE (BOX SPANNING THE 25TH TO 75TH PERCENTILES). THE UPPER WHISKER EXTENDS FROM THE HINGE TO THE HIGHEST VALUE THAT IS WITHIN 1.5 * IQR OF THE HINGE, WHERE IQR IS THE INTER-QUARTILE RANGE. THE LOWER WHISKER EXTENDS FROM THE HINGE TO THE LOWEST VALUE WITHIN 1.5 * IQR OF THE HINGE. 5

FIGURE S 4. THE AGE-TRENDS IN SCFA AND BA LEVELS WERE SIMILAR IN THE SUBSET OF CHILDREN WITH THE WHOLE TIMESERIES AVAILABLE, AND BREASTFEEDING COVARIED WITH THE AGE, RELATED TO FIGURE 1. 6

FIGURE S 5. CONTRIBUTION OF TAXONOMIC GROUPS TO THE DMM CLUSTERS, RELATED TO FIGURE 3. 7

FIGURE S 6 DIFFERENCES IN DEMOGRAPHIC FACTORS IN CLUSTERS PER TIMEPOINT. THE BARS REPRESENT NUMBER OF INDIVIDUALS. FINDINGS WITH P-VALUE <0.05 ARE VISUALIZED, RELATED TO FIGURE 3. 9

FIGURE S 7. ALDEX2 INDICATED MULTIPLE ASSOCIATIONS BETWEEN BILE ACIDS CONCENTRATIONS AND BIFIDOBACTERIUM AND CLOSTRIDIUM ABUNDANCES AT 2.5 MO. INTERESTINGLY, THEY WERE IN OPPOSING DIRECTIONS. BILE ACID DATA IS LOG-TRANSFORMED WHEREAS ABUNDANCE DATA IS ROBUST CENTERED LOG-TRANSFORMED (RCLR) FOR THE VISUALIZATION, RELATED TO FIGURE 4. 11

FIGURE S 8. AT 30 MONTHS UNIDENTIFIED GENUS IN OSCILLOSPIRALES ORDER ASSOCIATED NEGATIVE WITH BILE ACIDS. BILE ACID DATA IS LOG-TRANSFORMED WHEREAS ABUNDANCE DATA IS ROBUST CENTERED LOG-TRANSFORMED (RCLR) FOR THE VISUALIZATION, RELATED TO FIGURE 4. 12

FIGURE S 9. THE BA AND SCFA LEVEL DIFFERENCES BETWEEN CLUSTERS WERE SIMILAR IN THE SUBSET OF CHILDREN WITH THE WHOLE TIMESERIES AVAILABLE, RELATED TO FIGURE 6. A., B. EACH BOX IN THE PLOT SHOWS THE MEDIAN (HORIZONTAL LINE), AND THE INTERQUARTILE RANGE (BOX SPANNING THE 25TH TO 75TH PERCENTILES). THE UPPER WHISKER EXTENDS FROM THE HINGE TO THE

HIGHEST VALUE THAT IS WITHIN 1.5 * IQR OF THE HINGE, WHERE IQR IS THE INTER-QUARTILE RANGE. THE LOWER WHISKER EXTENDS FROM THE HINGE TO THE LOWEST VALUE WITHIN 1.5 * IQR OF THE HINGE 13
FIGURE S 10. DURATION OF EXCLUSIVE BREASTFEEDING AND INTERACTION WITH BACTEROIDES AND ESCHERICHIA INTERACTION ASSOCIATED WITH 7-OXO-CONVERTED BA. MEAN SPLIT FOR THE DURATION OF EXCLUSIVE BREASTFEEDING IS USED HERE FOR ILLUSTRATION PURPOSES, ALTHOUGH CONTINOUS VARIABLE WAS USED IN THE ANALYSES. MODEL FORMULA WAS METABOLITE CONCENTRATION ~ DURATION OF EXCLUSIVE BREASTFEEDING + AGE + ANY CURRENT BREASTFEEDING + 1|ID, RELATED TO FIGURE 7. GREY AREAS DEPICT 95 % CONFIDENCE INTERVALS. 14
FIGURE S 11. POSITIVE AND NEGATIVE CONTROL SAMPLES IN 2.5 MONTHS TIME POINT. A. READ COUNTS ACROSS CONTROL SAMPLES. B. RELATIVE ABUNDANCES OF CORE GENERA IN POSITIVE CONTROL SAMPLES, RELATED TO STAR METHODS, METHOD DETAILS. 15
FIGURE S 12. POSITIVE AND NEGATIVE CONTROL SAMPLES IN 6 MONTHS TIME POINT. A. READ COUNTS ACROSS CONTROL SAMPLES. B. RELATIVE ABUNDANCES OF CORE GENERA IN POSITIVE CONTROL SAMPLES, RELATED TO STAR METHODS, METHOD DETAILS. 16
FIGURE S 13. POSITIVE AND NEGATIVE CONTROL SAMPLES IN 14 MONTHS TIME POINT. A. READ COUNTS ACROSS CONTROL SAMPLES PER CONTROL SAMPLE TYPE. B. READ COUNTS IN ALL INDIVIDUAL CONTROL SAMPLES. C. RELATIVE ABUNDANCES OF CORE GENERA IN POSITIVE CONTROL SAMPLES, RELATED TO STAR METHODS, METHOD DETAILS. 17
FIGURE S 14. POSITIVE AND NEGATIVE CONTROL SAMPLES IN 30 MONTHS TIME POINT. A. READ COUNTS ACROSS CONTROL SAMPLES. B. RELATIVE ABUNDANCES OF CORE GENERA IN POSITIVE CONTROL SAMPLES, RELATED TO STAR METHODS, METHOD DETAILS. 18
FIGURE S 15. LIBRARY SIZES IN ALL TIMEPOINTS AND THE WHOLE SAMPLE, RELATED TO STAR METHODS, METHOD DETAILS. EACH BOX IN THE PLOT SHOWS THE MEDIAN (HORIZONTAL LINE), AND THE INTERQUARTILE RANGE (BOX SPANNING THE 25TH TO 75TH PERCENTILES). THE UPPER WHISKER EXTENDS FROM THE HINGE TO THE HIGHEST VALUE THAT IS WITHIN 1.5 * IQR OF THE HINGE, WHERE IQR IS THE INTER-QUARTILE RANGE. THE LOWER WHISKER EXTENDS FROM THE HINGE TO THE LOWEST VALUE WITHIN 1.5 * IQR OF THE HINGE. 20

Supplementary tables and pages:

TABLE S 2. DEMOGRAPHIC FACTORS ASSOCIATED WITH CLUSTER MEMBERSHIP PER TIMEPOINT, RELATED TO FIGURE 3. 9
TABLE S 3. DUNN'S POSTHOC TEST FOR SIGNIFICANT CLUSTER DIFFERENCES IN TIMEPOINT-WISE GROUP COMPARISON, RELATED TO FIGURE 3 9
TABLE S 4. CLUSTER PI AND THETA VALUES. CLUSTERS DIFFERED BY THE VARIABILITY (THETA). LOWER THETA VALUES INDICATE HIGHER VARIANCE, AND THE C5 AND C7 HAVE THE MOST VARIANCE IN THE TAXONOMIC CONTRIBUTIONS. MOREOVER, THE C1 SEEMS TO HAVE HIGHER VARIANCE COMPARED WITH C2 AND C3, RELATED TO FIGURE 3. 10

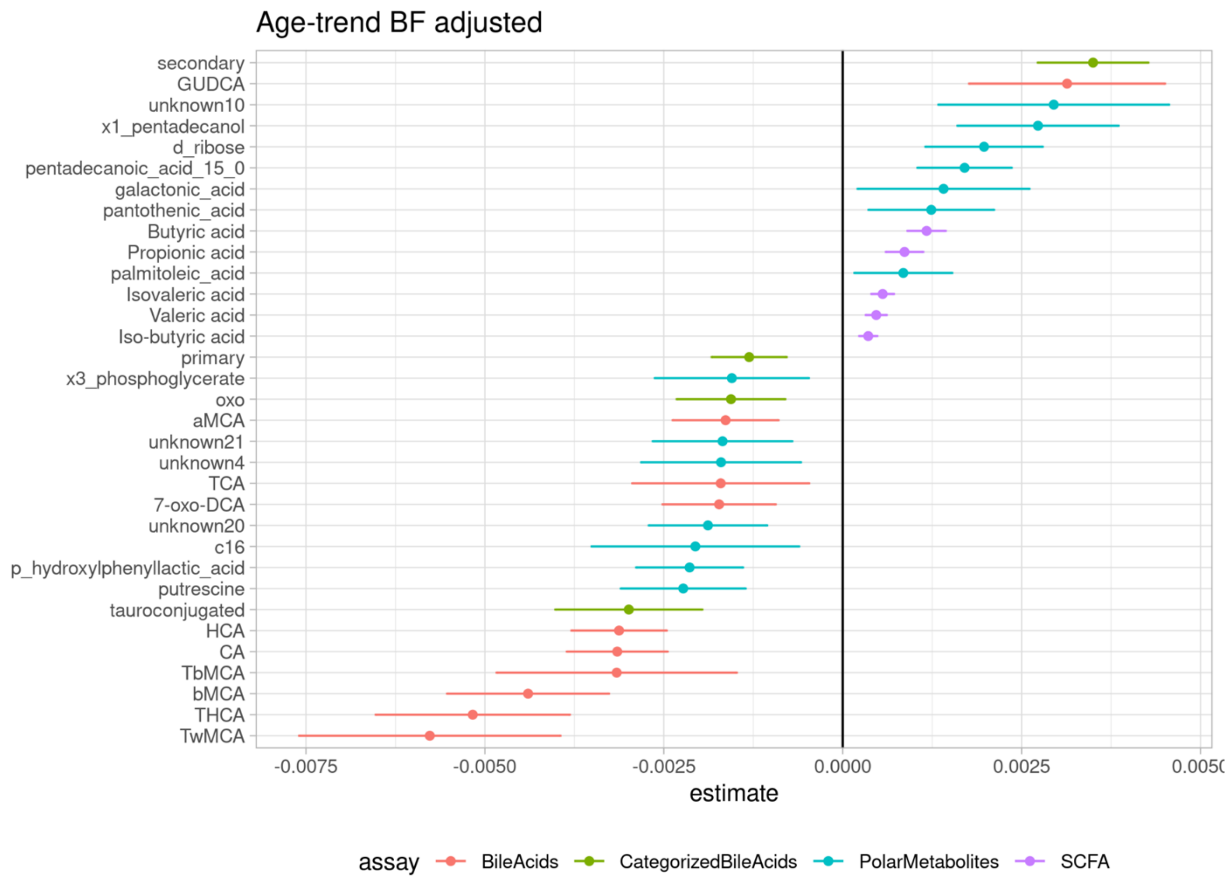

Figure S 1Estimates and 95 % confidence intervals for metabolites associated with age when adjusting for current breastfeeding, related to Figure 1. Error bars represent 95% confidence intervals.

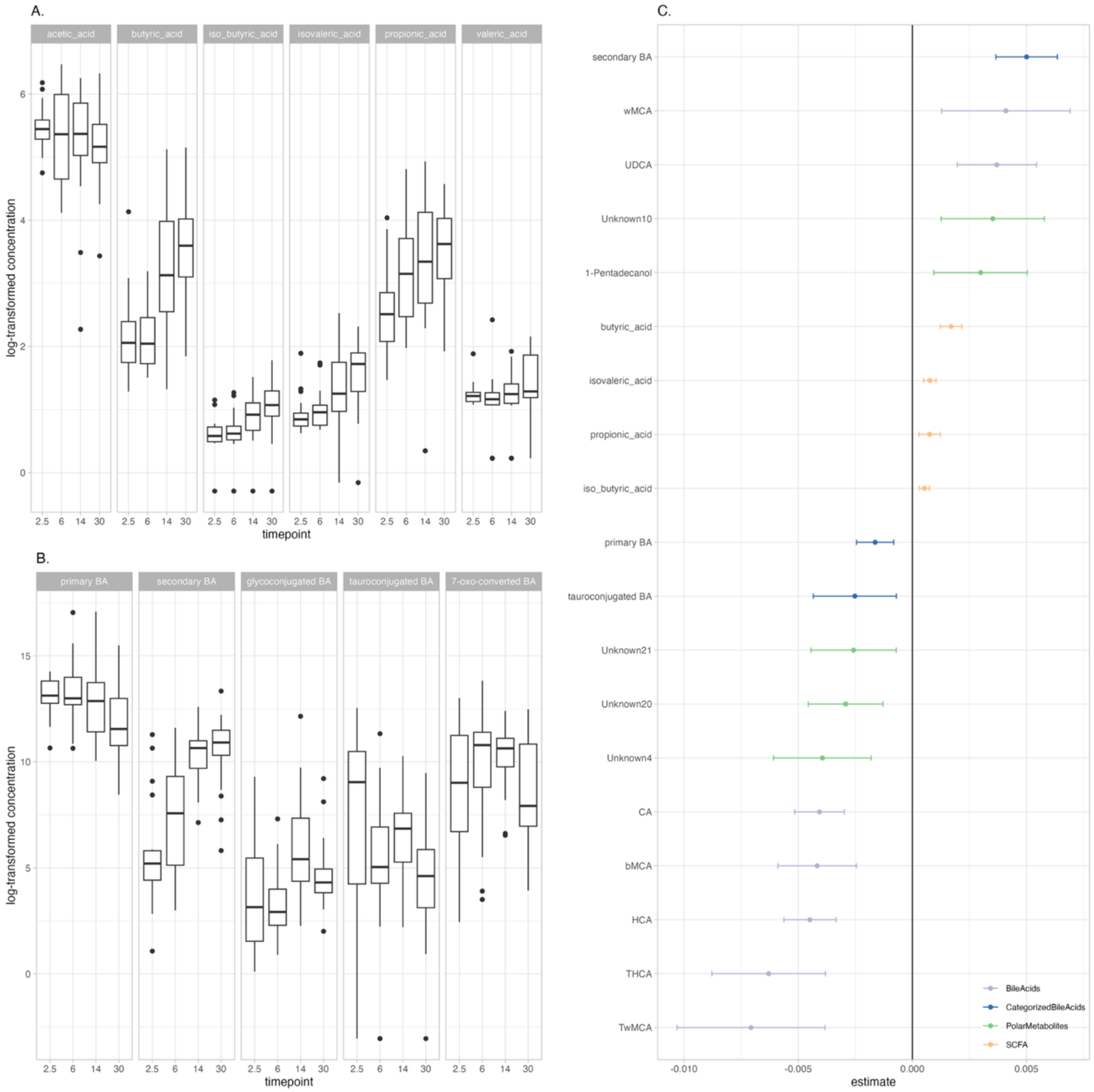

*Figure S 2 Metabolite age-trends in the subsample of children with the whole timeseries, related to Figure 1. A., B. Each box in the plot shows the median (horizontal line), and the interquartile range (box spanning the 25th to 75th percentiles). The upper whisker extends from the hinge to the highest value that is within 1.5 * IQR of the hinge, where IQR is the inter-quartile range. The lower whisker extends from the hinge to the lowest value within 1.5 * IQR of the hinge. C. Error bars represent 95% confidence intervals.*

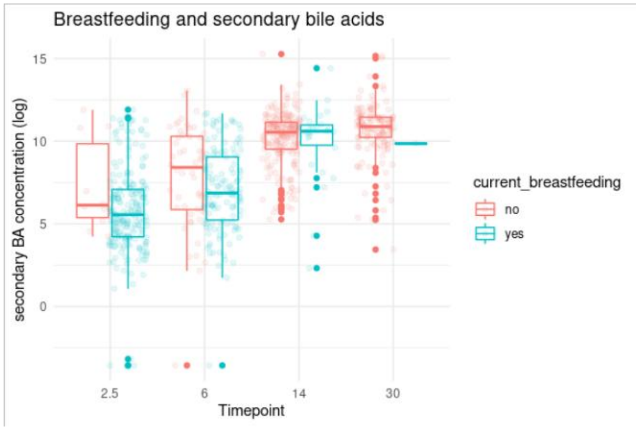

*Figure S 3 Secondary bile acid concentrations in breastfed and non-breastfed children per timepoint, related to Figure 2. Each box in the plot shows the median (horizontal line), and the interquartile range (box spanning the 25th to 75th percentiles). The upper whisker extends from the hinge to the highest value that is within 1.5 * IQR of the hinge, where IQR is the inter-quartile range. The lower whisker extends from the hinge to the lowest value within 1.5 * IQR of the hinge.*

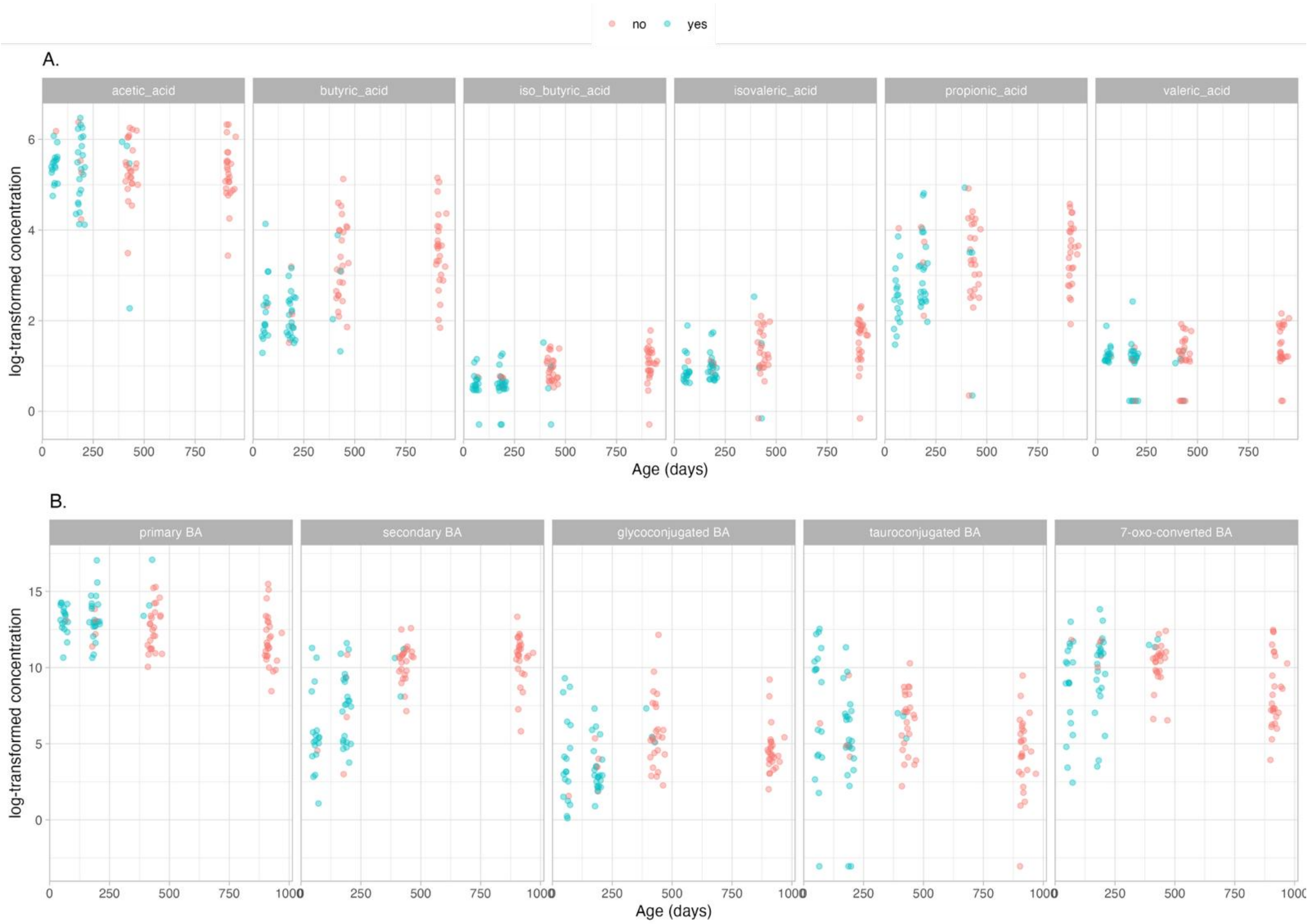

Figure S 4. The age-trends in SCFA and BA levels were similar in the subset of children with the whole timeseries available, and breastfeeding covaried with the age, related to Figure 1.

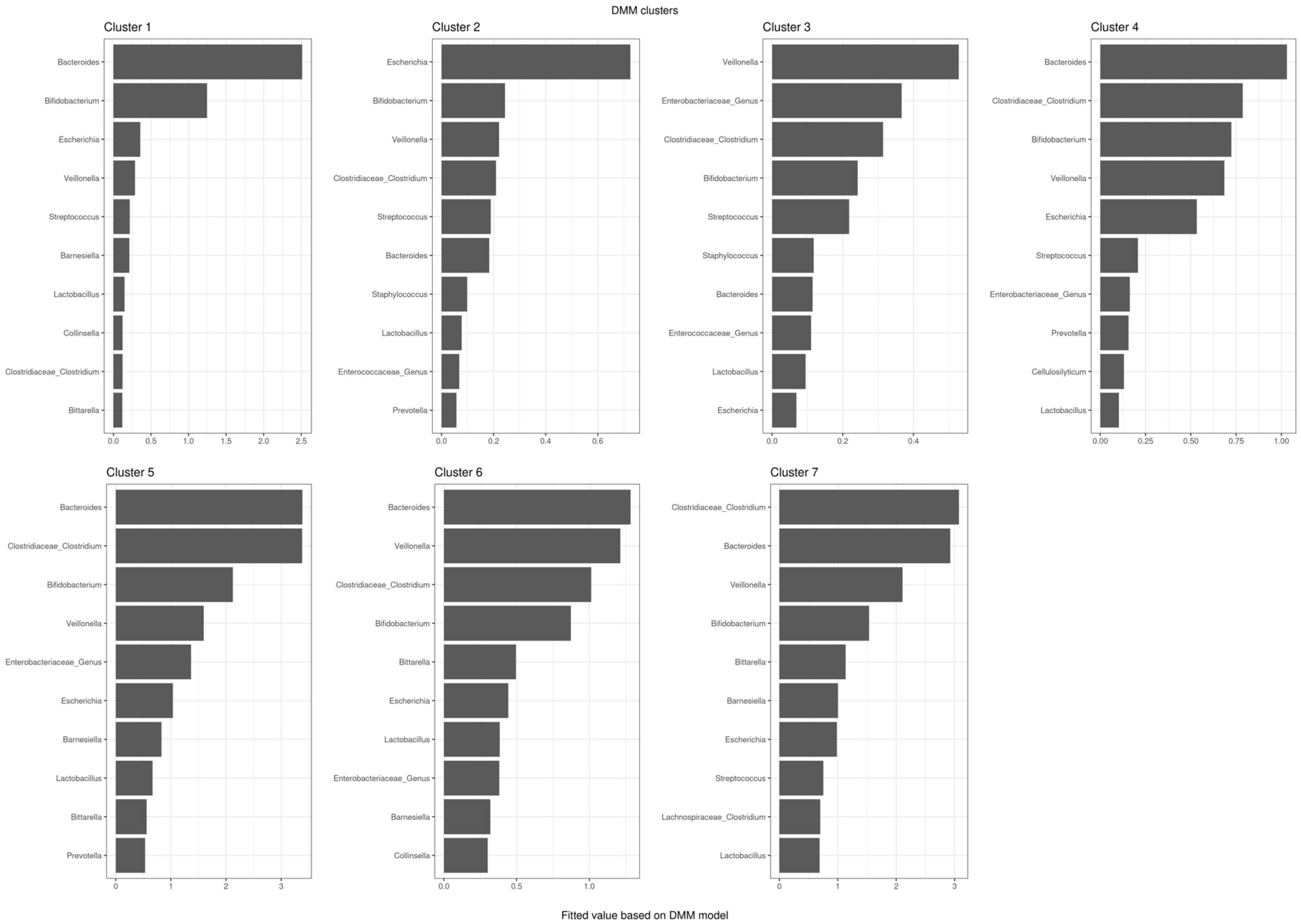

Figure S 5. Contribution of taxonomic groups to the DMM clusters, related to Figure 3.

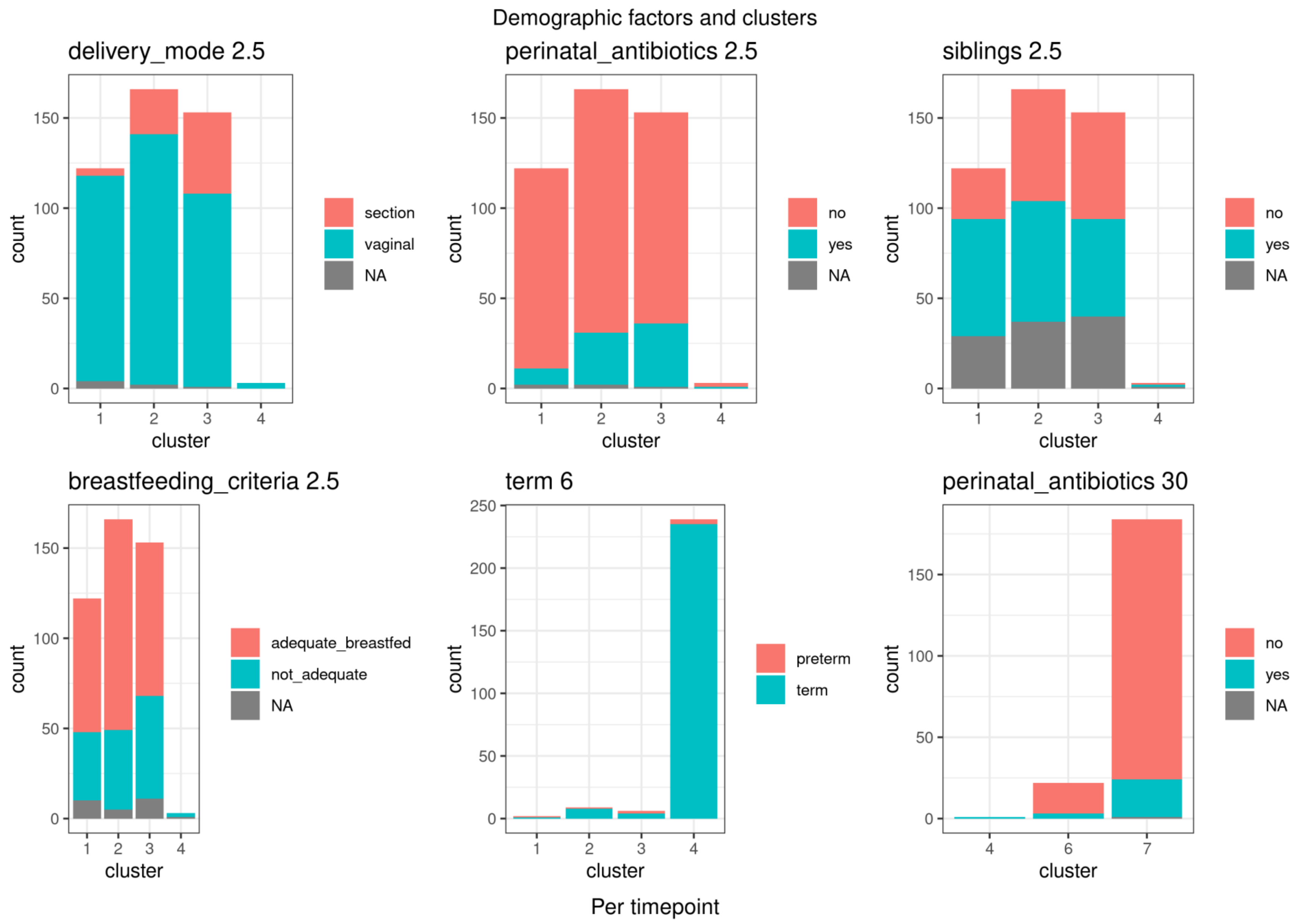
Demographic factors and clusters
delivery_mode 2.5
section
vaginal
NA
perinatal_antibiotics 2.5
no
yes
NA
siblings 2.5
no
yes
NA
breastfeeding_criteria 2.5
adequate_breastfed
not_adequate
NA
term 6
preterm
term
perinatal_antibiotics 30
no
yes
NA
count
cluster
Per timepoint

Figure S 6 Differences in demographic factors in clusters per timepoint. The bars represent number of individuals. Findings with p-value <0.05 are visualized, related to Figure 3.

Table S 2. Demographic factors associated with cluster membership per timepoint, related to Figure 3.

timepoint	var	statistic	chisq_df	p_value	dunns_groups	q
6	term	36.2	3	0.00E+00	4.00E+00	0
2.5	delivery_mode	33.7	3	0.00E+00	3.00E+00	0
2.5	perinatal_antibiotics	12.3	3	0.00647	1	0.0733
2.5	siblings	11.2	3	0.01065	2	0.09054
2.5	breastfeeding_criteria	9.5	3.00E+00	0.02354	0	0.16007
30	perinatal_antibiotics	6.7	2	0.03541	2	0.20064

Table S 3. Dunn's posthoc test for significant cluster differences in timepoint-wise group comparison, related to Figure 3

timepoint	var	clusters	p.adj	statistic
2.5	delivery_mode	1 vs 2	0.03566	-2.60E+00
2.5	delivery_mode	1 vs 3	0	-5.70E+00
2.5	delivery_mode	2 vs 3	0.0034	-3.4
6	term	1 vs 2	0.01729	2.9

6	term	1 vs 4	4.70E-04	3.9
6	term	2 vs 3	0.04675	-2.4
6	term	3 vs 4	7.00E-05	4.4

Table S 4. Cluster pi and theta values. Clusters differed by the variability (theta). Lower theta values indicate higher variance, and the C5 and C7 have the most variance in the taxonomic contributions. Moreover, the C1 seems to have higher variance compared with C2 and C3, related to Figure 3.

cluster	pi	theta
1	0.1	6.48
2	0.14	2.48
3	0.14	2.73
4	0.2	5.73
5	0.09	23.67
6	0.17	10.54
7	0.15	25.67

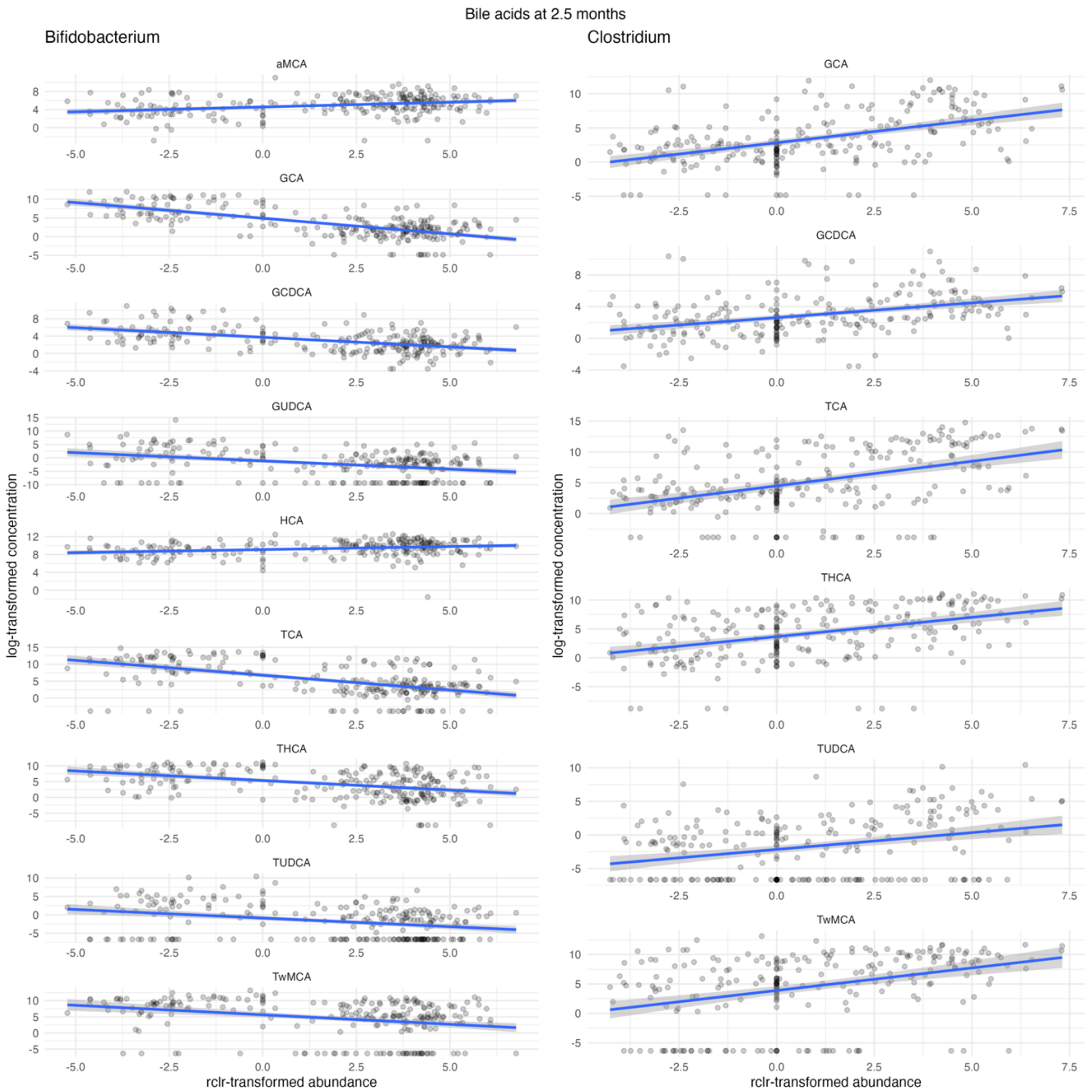

Figure S 7. ALDEx2 indicated multiple associations between bile acids concentrations and Bifidobacterium and Clostridium abundances at 2.5 mo. Interestingly, they were in opposing directions. Bile acid data is log-transformed whereas abundance data is robust centered log-transformed (rclr) for the visualization, related to Figure 4.

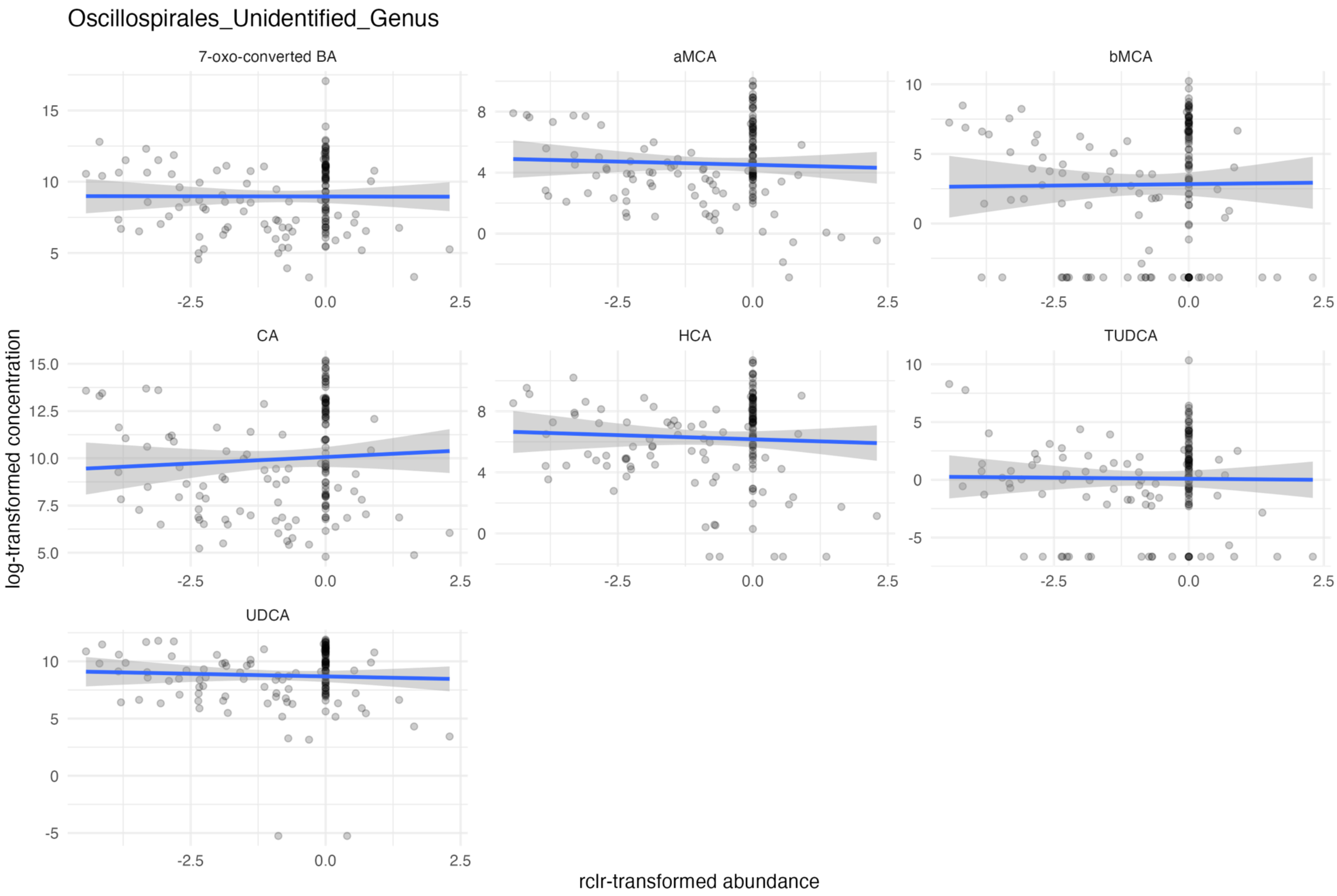

Figure S 8. At 30 months unidentified genus in Oscillospirales order associated negative with bile acids. Bile acid data is log-transformed whereas abundance data is robust centered log-transformed (rclr) for the visualization, related to Figure 4.

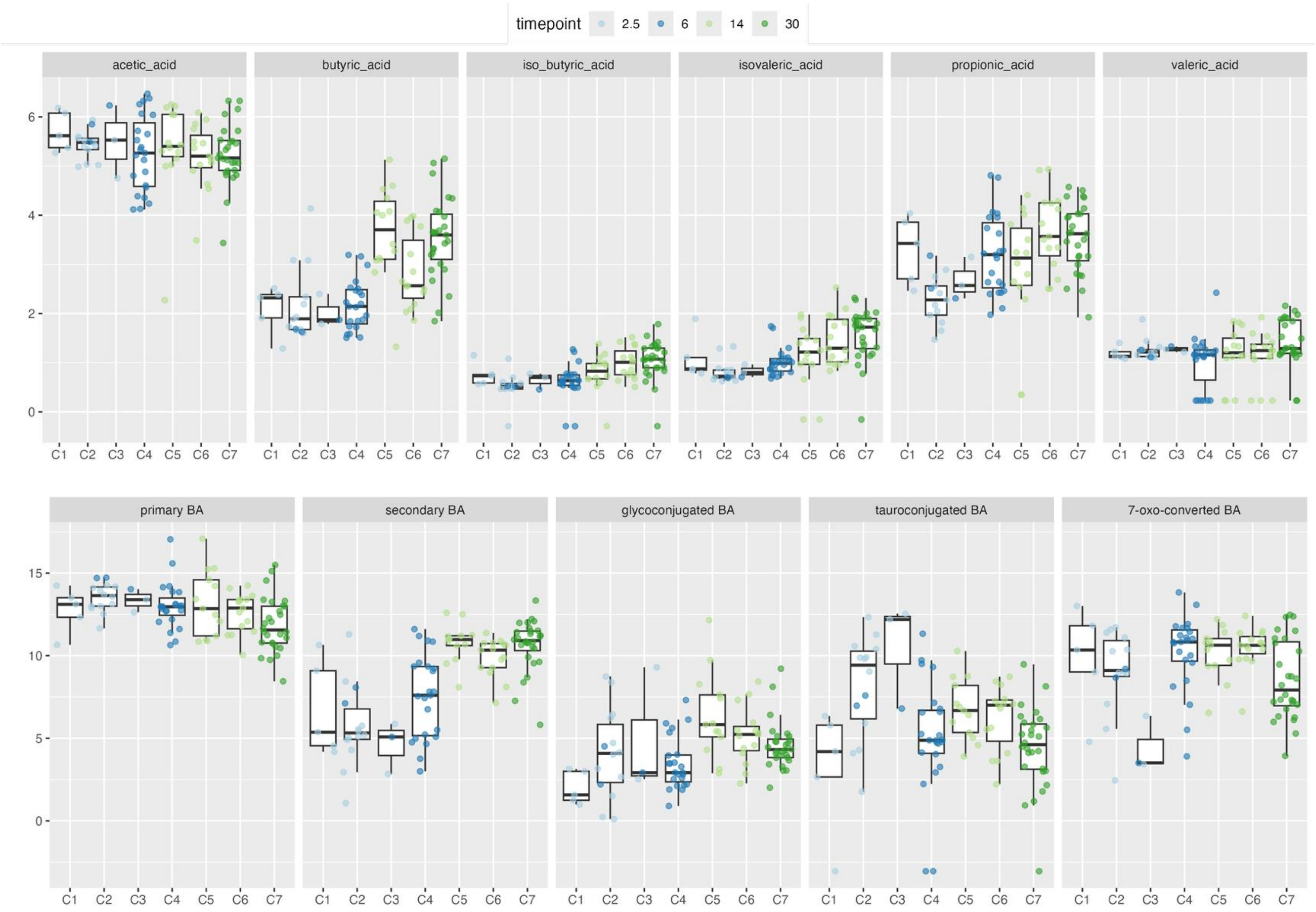

*Figure S 9. The BA and SCFA level differences between clusters were similar in the subset of children with the whole timeseries available, related to Figure 6. A., B. Each box in the plot shows the median (horizontal line), and the interquartile range (box spanning the 25th to 75th percentiles). The upper whisker extends from the hinge to the highest value that is within 1.5 * IQR of the hinge, where IQR is the inter-quartile range. The lower whisker extends from the hinge to the lowest value within 1.5 * IQR of the hinge*

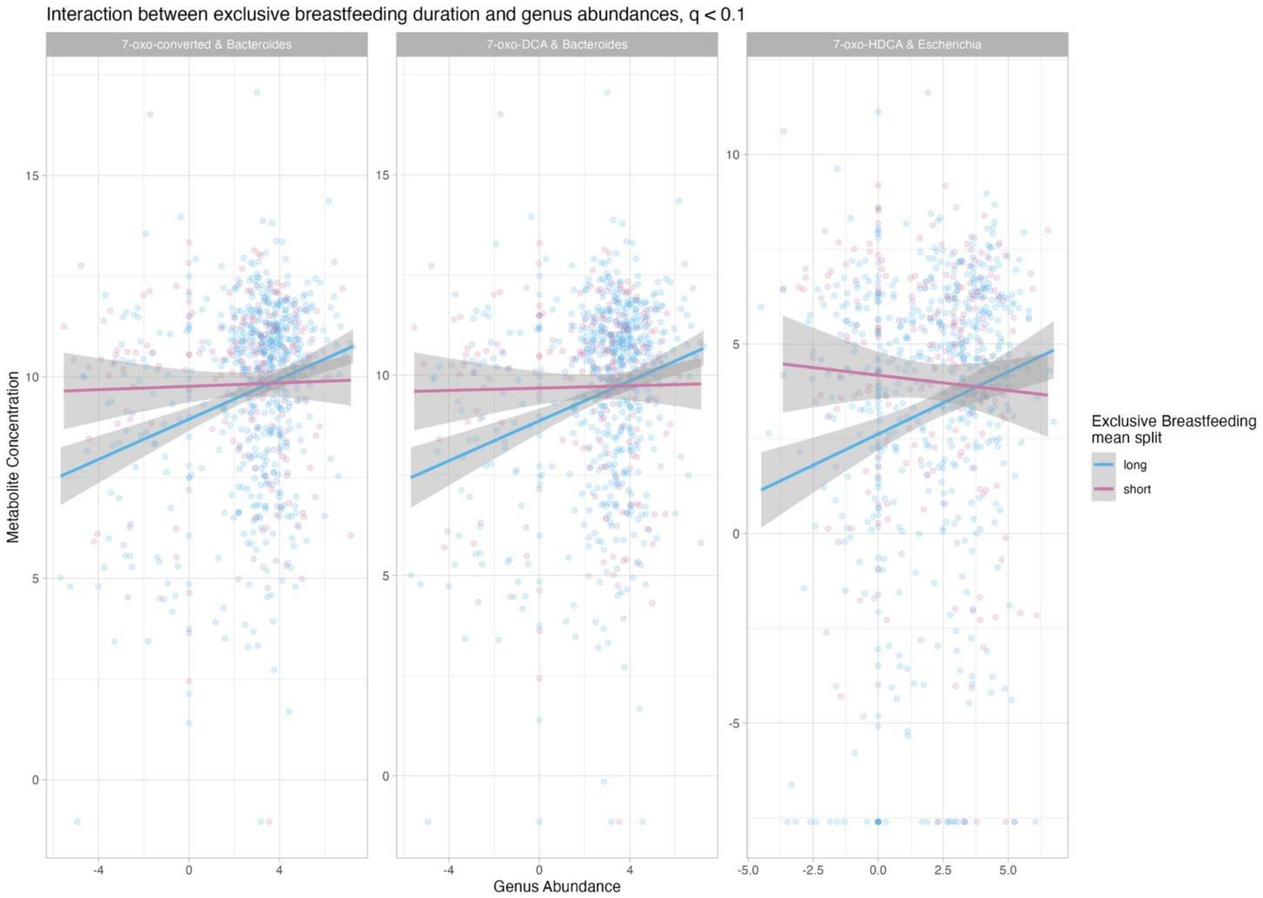

Figure S 10. Duration of exclusive breastfeeding and interaction with Bacteroides and Escherichia interaction associated with 7-oxo-converted BA. Mean split for the duration of exclusive breastfeeding is used here for illustration purposes, although continous variable was used in the analyses. Model formula was metabolite concentration ~ duration of exclusive breastfeeding + age + any current breastfeeding + 1|ID, related to Figure 7. Grey areas depict 95 % confidence intervals.

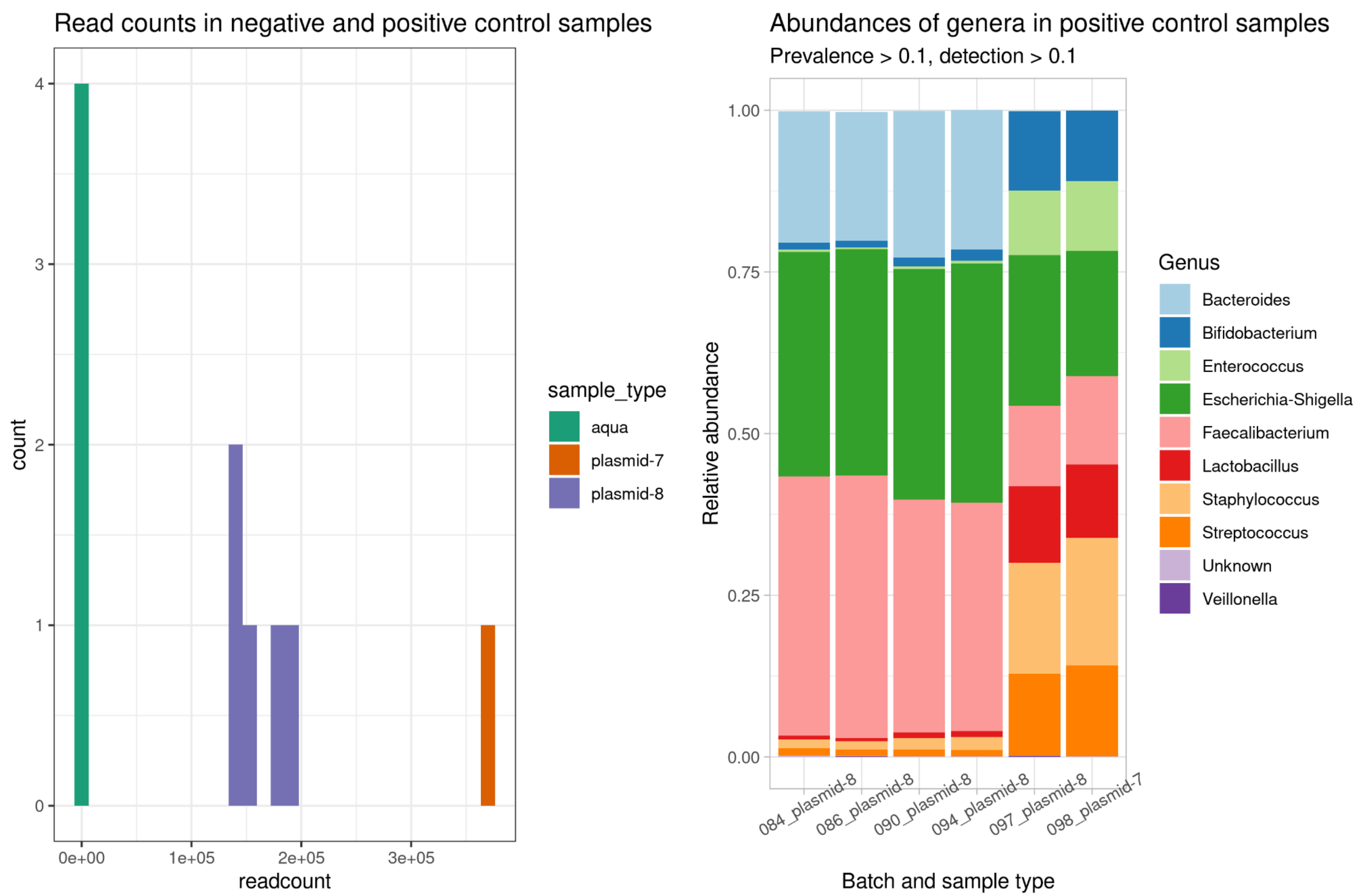

Figure S 11. Positive and negative control samples in 2.5 months time point. A. read counts across control samples. B. Relative abundances of core genera in positive control samples, related to STAR Methods, Method Details.

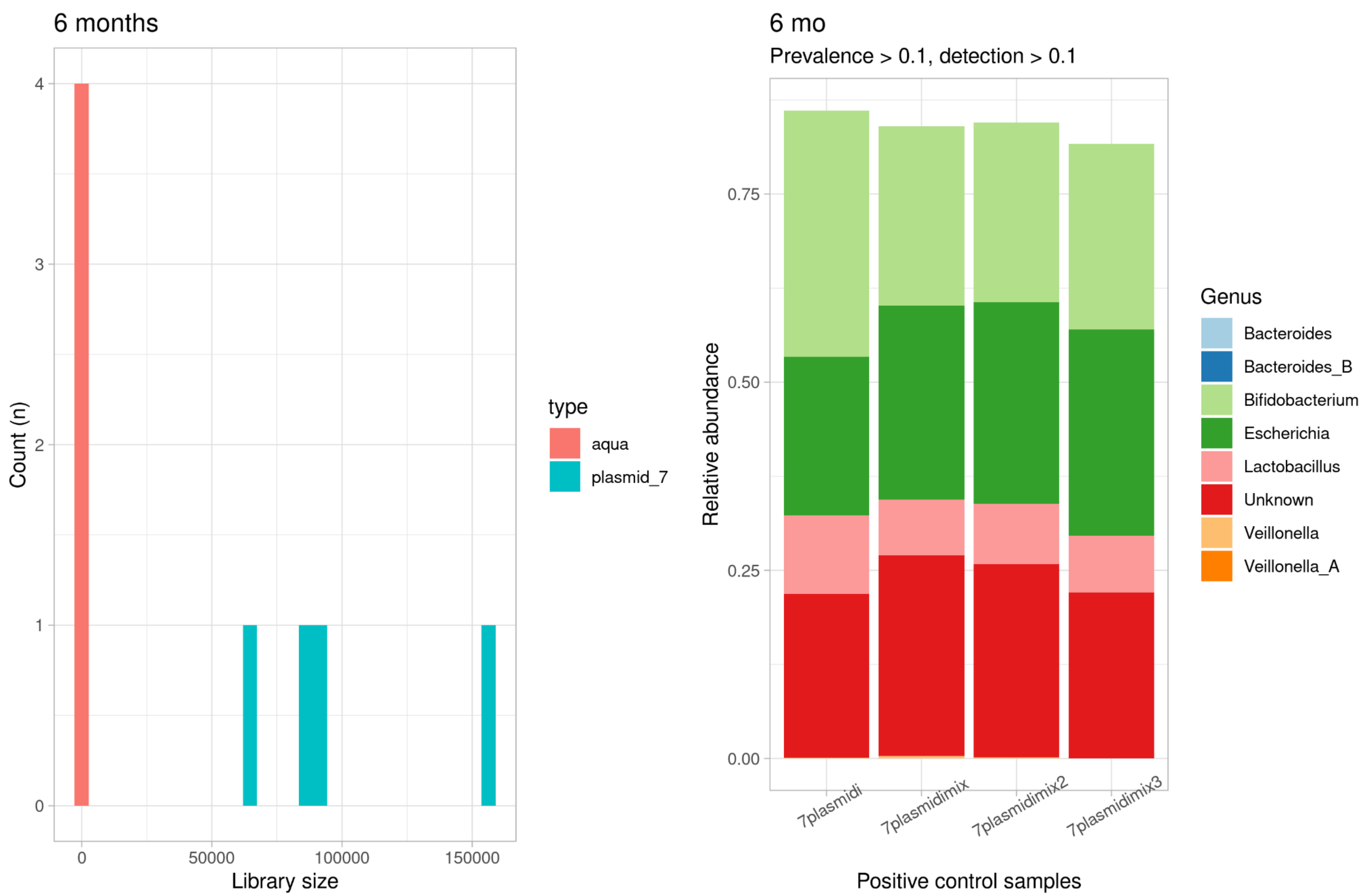

Figure S 12. Positive and negative control samples in 6 months time point. A. read counts across control samples. B. Relative abundances of core genera in positive control samples, related to STAR Methods, Method Details.

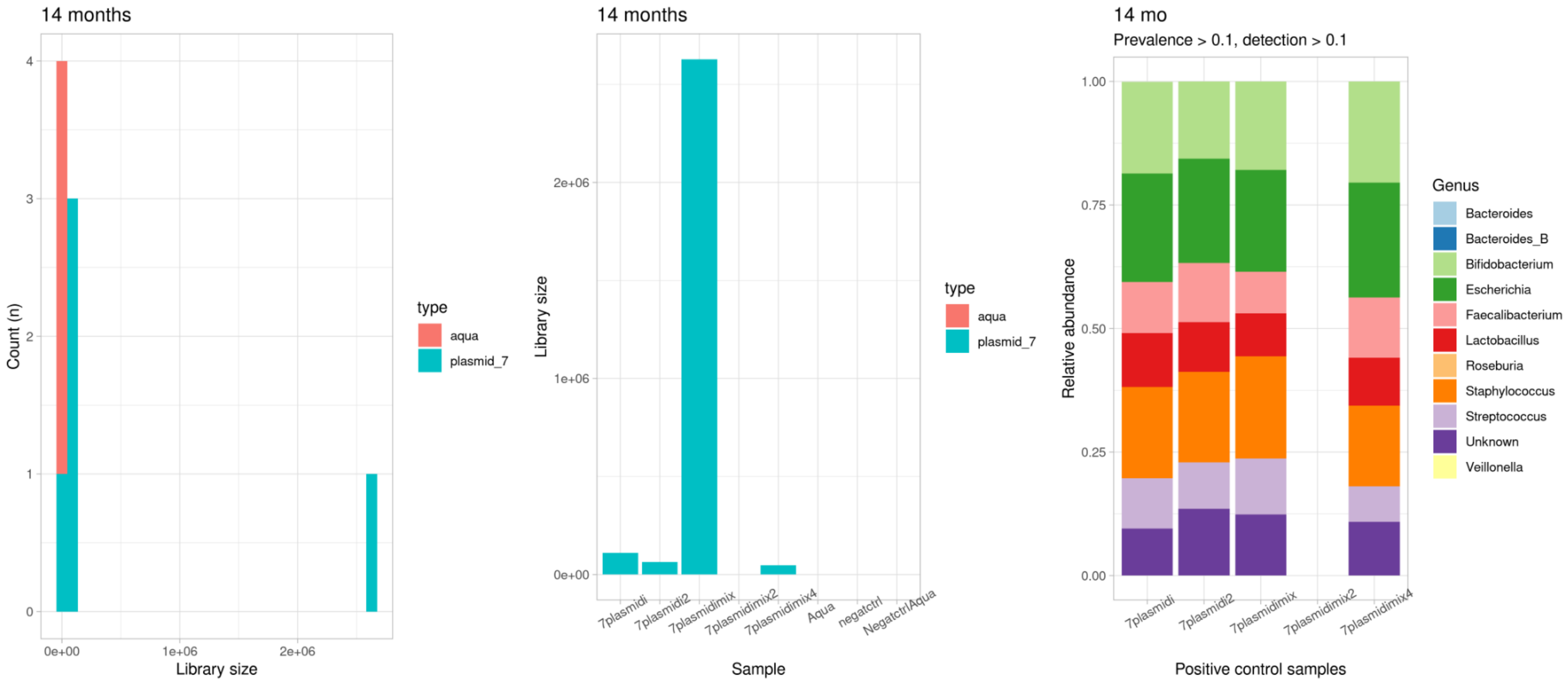

Figure S 13. Positive and negative control samples in 14 months time point. A. read counts across control samples per control sample type. B. Read counts in all individual control samples. C. Relative abundances of core genera in positive control samples, related to STAR Methods, Method Details.

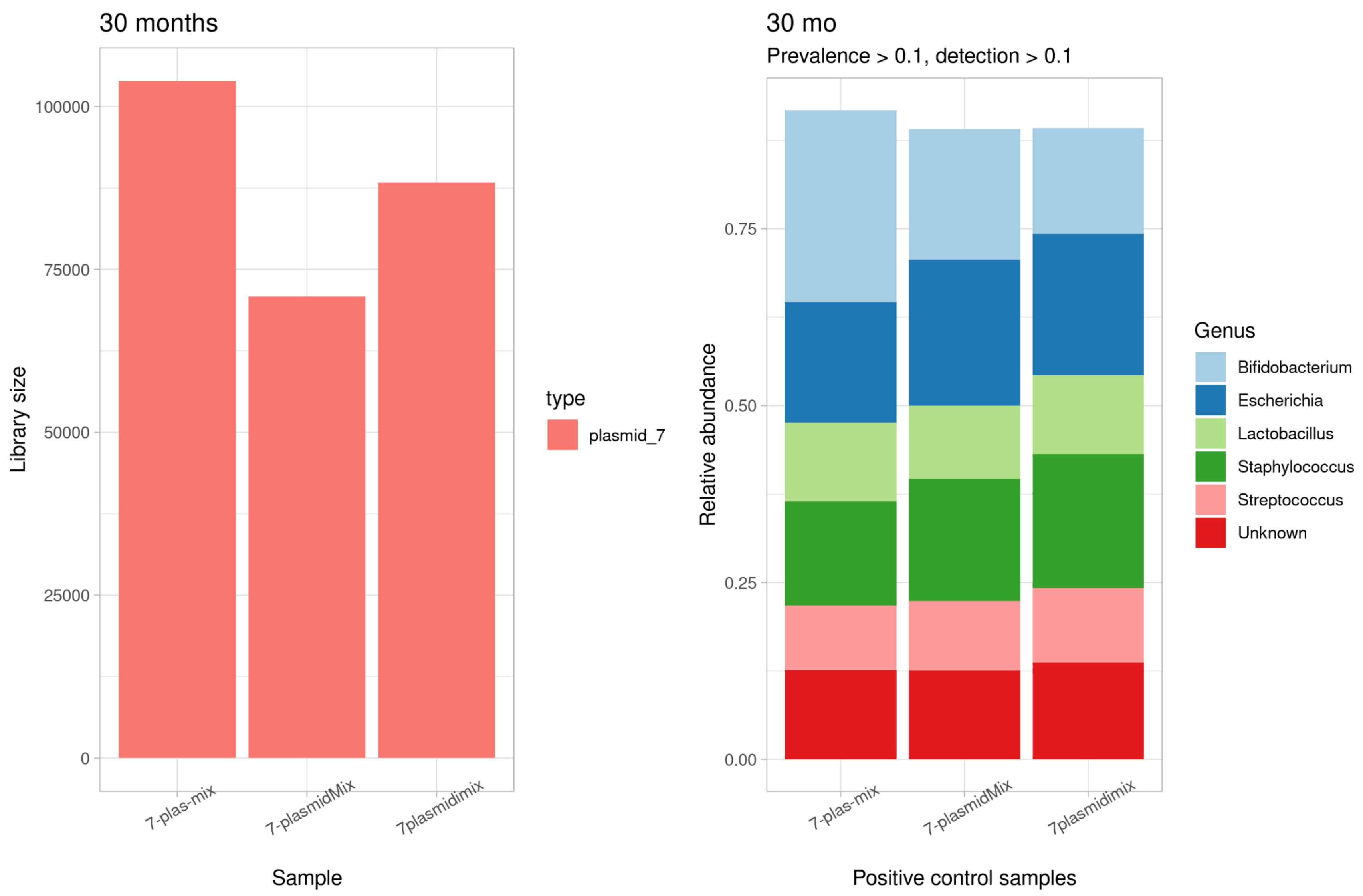

Figure S 14. Positive and negative control samples in 30 months time point. A. read counts across control samples. B. Relative abundances of core genera in positive control samples, related to STAR Methods, Method Details.

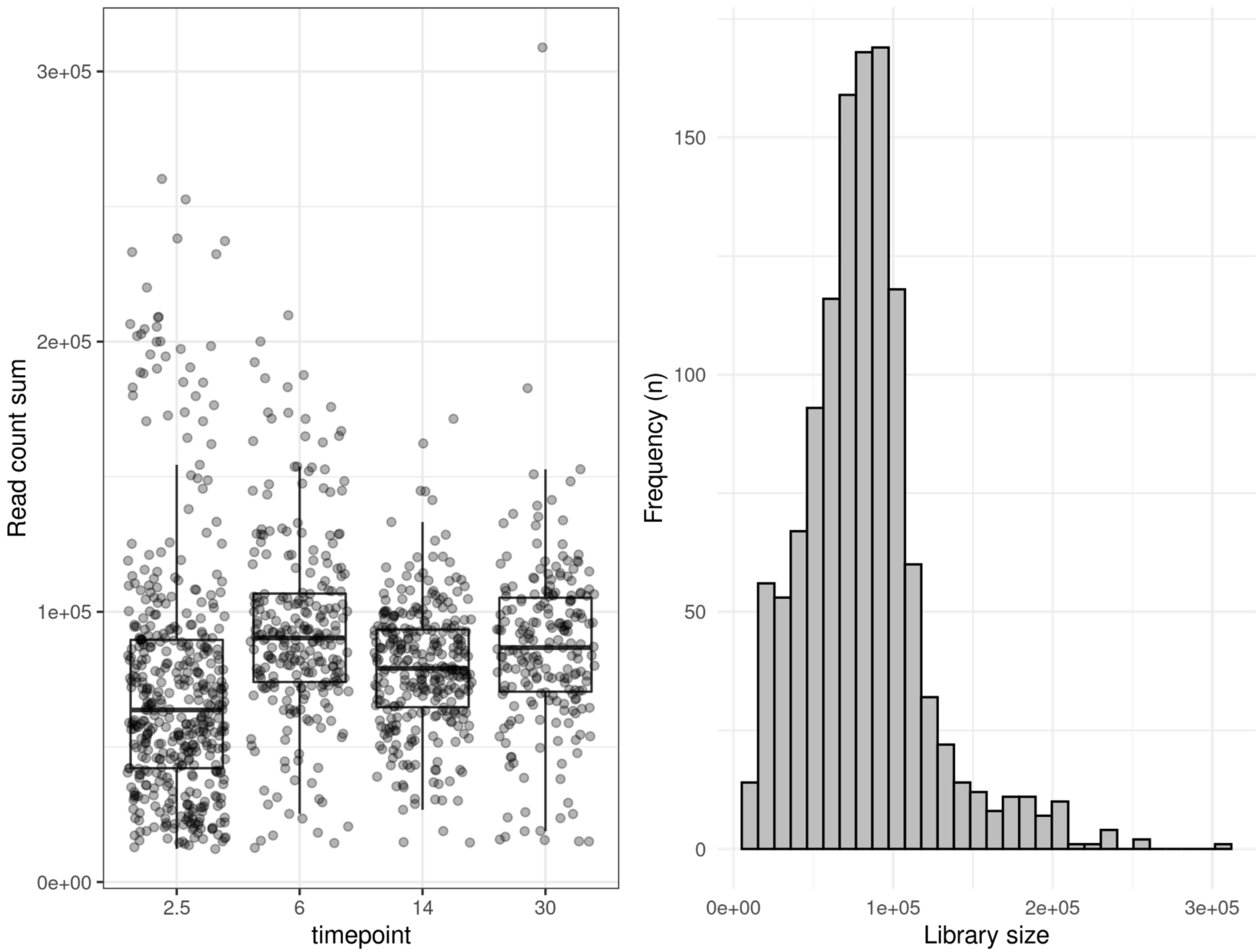

Figure S 15. Library sizes in all timepoints and the whole sample, related to STAR Methods, Method Details. Each box in the plot shows the median (horizontal line), and the interquartile range (box spanning the 25th to 75th percentiles). The upper whisker

extends from the hinge to the highest value that is within 1.5 * IQR of the hinge, where IQR is the inter-quartile range. The lower whisker extends from the hinge to the lowest value within 1.5 * IQR of the hinge.
